# Pre-harvest cinnamic acid and potassium cinnamate improve postharvest quality and storability of ‘Kyoho’ grapes

**DOI:** 10.3389/fpls.2025.1713059

**Published:** 2026-01-12

**Authors:** Yulong Hu, Chaoxia Wang, Shufen Tian, Rong Wang, Chuang Ma, Jianfu Jiang

**Affiliations:** College of Horticulture and Landscape Architecture, Tianjin Agricultural University, Tianjin, China

**Keywords:** cinnamic acid, entropy weight TOPSIS, fruit quality, ‘Kyoho’ grape, potassium cinnamate, storability

## Abstract

Frequent high temperatures render ‘Kyoho’ grapes prone to postharvest softening, berry drop, and insufficient coloration. To address these challenges, this study investigated the effects of preharvest foliar sprays of cinnamic acid (CA) and potassium cinnamate (PC) on fruit quality and storability. Grapes were treated with different concentrations of CA or PC from veraison to maturity, and followed by evaluation after 96 hours of storage at 25°C. The results indicated that high concentrations of CA and PC significantly enhanced fruit quality by increasing total soluble solids (TSS) content and the accumulation of antioxidants (e.g., total phenols, flavonoids, and ascorbic acid), while reducing titratable acidity (TA) and improving texture properties including peel puncture strength, flesh firmness, and elasticity. Furthermore, these treatments inhibited hydrolase activity (e.g., cellulase and pectinase), decreased berry drop and decay incidence, and suppressed pathogen growth (especially Penicillium and Aspergillus). Notably, 10 mmol·L^-1^ CA was most effective in maintaining TSS and antioxidant reserves, whereas 10 mmol·L^-1^ PC excelled in preserving fruit firmness, improving pedicel stability, and extending storage life. These beneficial effects were mediated by regulating the activity of key enzymes (e.g., SOD, POD, PPO, C4H, and 4CL) and the expression of relevant genes (e.g., *VvPAL*, *VvCHS*, and *VvANS*), thereby providing a practical technical foundation for the postharvest preservation of ‘Kyoho’ grapes.

## 1 Introduction

‘Kyoho’ grape, are tetraploid varieties native to Japan, developed as a crossbreed of *Vitis vinifera* (European grape) and *V. labrusca* (American grape). Renowned for their large berries, juicy flesh, unique flavor, and high sugar content (≥18%), they are widely favored in East Asia and globally, holding significant economic value (Chen, 2019). In the context of global warming, frequent extreme high temperatures during the ripening stage trigger severe postharvest deterioration. This includes hastened cell wall degradation, disruption of the antioxidant system homeostasis, and premature formation of the abscission layer in the pedicel (Pablo et al., 2013). These lead to berry softening, pre-harvest berry drop, and shortened shelf life (Zhang, 2020). Moreover, high-temperatures and humidity environments create favorable conditions for fungal infections, such as gray mold and anthracnose, resulting in postharvest losses of 30% - 45% and severely restricting the sustainable development of the grape industry (Tyagi et al., 2020).

Currently, postharvest preservation methods primarily depend on regulators, such as abscisic acid (ABA) and salicylic acid; however, these substances frequently present environmental risks or incur high costs. Cinnamic acid (CA) and its potassium salt potassium cinnamate (PC), as natural phenolic compounds, provide the benefits of environmental friendliness and low cost, and have been proven to possess plant growth regulatory and antibacterial activities (Ruwizhi and Aderibigbe, 2020). As essential intermediates in the phenylpropanoid pathway, CA can modulate the expression of genes related to anthocyanin biosynthesis and lignin biosynthesis-related genes (Shang et al., 2022). Additionally, CA enhances the activity of antioxidant enzymes and inhibits plant pathogens such as *Botrytis cinerea* (Wang et al., 2021). However, the concentration-dependent effects of pre-harvest CA/PC application on grape quality and storability, as well as their synergistic regulatory mechanisms under high-temperature stress, remain poorly understood.

Based on previous research, we hypothesized that pre-harvest application of CA/PC would alleviate postharvest deterioration of grape by regulating the phenylpropanoid pathway, strengthening antioxidant defense systems, and stabilizing cell walls. Specifically, high concentrations of CA would focus on preserving fruit quality and antioxidant reserves, whereas PC would be particularly effective in enhancing berry firmness and disease resistance. This study systematically evaluated the effects of different CA/PC concentrations on ‘Kyoho’ grapes, analyzing metabolite accumulation, key enzyme activities, and the expression of genes associated with anthocyanin and lignin biosynthesis. The objective was to establish a plant-derived substance-based strategy for the prevention and control of high-temperature stress, thereby providing theoretical support for the grape industry in addressing climate change.

## 2 Materials and methods

### 2.1 Test materials and treatments

The experiment was conducted at Tianjin Binhai Chadian Grape Science and Technology Park (E117.788952, N39.251031°). The soil at the experimental base was coastal fluvo-aquic, and the experimental materials were 10-year-old self-rooted seedlings of ‘Kyoho’ grapes cultivated in a multi-span plastic greenhouse, using single-trunk trellis cultivation (trellis height 2 m). The trellis was oriented east-west, with a plant spacing of 1.5 m × 4.5 m. The phenological periods and vegetative growth status of grapevines and cultivation management techniques were consistent across all plants.

The field experiment comprised eight treatments (see **Table 1**): CK served as the distilled water control; T0 contained 10 mmol·L^-1^ hydroxypropyl-β-cyclodextrin (HP-β-CD); T1, T2, and T3 represented mixtures of 1, 5, 10 mmol·L^-1^ CA and 10 mmol·L^-1^ HP-β-CD solution, respectively; T4, T5, and T6 were 1, 5, and 10 mmol·L^-1^ PC, respectively. HP-β-CD is a commonly used solubilizer in plant-related studies. Preliminary tests confirmed that the solubilization ratio of HP-β-CD to CA is 1:1 (Koki et al., 2023), with the concentration of HP-β-CD determined based on the highest concentration of CA. Initial observations indicated that this concentration had no significant effect on core indicators such as the appearance and soluble solids of grape fruits. Due to experimental condition limitations, systematic validation was not performed; these are observational results. Concentrations of 1, 5, and 10 mmol·L^-1^ CA were determined based on existing research (Hu et al., 2024). This concentration range has been widely applied in studies on the effects of phenolic substances in grapes and similar fruits, covering low, medium, and high effective concentration gradients.

**Table 1:** Treatment nomenclature and supplies usage scale.

Treatments	HP-β-CD mmol·L^-1^	Cinnamic acid mmol·L^-1^	Potassium cinnamate mmol·L^-1^
CK	0	0	0
T0	10	0	0
T1	10	1	0
T2	10	5	0
T3	10	10	0
T4	0	0	1
T5	0	0	5
T6	0	0	10

A randomized complete block design was adopted for the experiment, establishing three blocks according to consistency in soil fertility and plant growth metrics (plant height, canopy width). Within each block, six grapevines per treatment were randomly selected as biological replicates, resulting in a total of 18 vines and 270 clusters (15 clusters per vine) for each treatment across the entire experiment. Cluster selection adhered to quantitative standards: cluster weight 140–160 g, number of berries per cluster 35 - 45, and berry skin color (no obvious color difference to the naked eye, CIE a* value: 4 - 6). To avoid subjective bias, target clusters were selected through random sampling. During sampling, measurement, and data recording, operators were blinded to the treatment groups, identifying samples only by code. To address pseudo-replication issues at the cluster level, blocks were treated as random effects in statistical models to control for variation between clusters.

Uniform spraying was performed on the marked clusters according to respective treatments at EL34 (45 days after flowering), EL35 (60 days after flowering), and EL38 (75 days after flowering) until the spray solution on the berry surface condensed into droplets and began to drip. Clusters were bagged after the surface droplets dried. Following the final treatment, grape berry maturity was monitored until the seeds turned brown and sugar content stabilized. Sampling was conducted on August 25, 2024, with 15 clusters collected from each treatment group and transported to Tianjin Agricultural University. Clusters were placed flat on an indoor laboratory bench (25 ± 1°C), and samples were taken at 0 h, 24 h, 48 h, 72 h, and 96 h to determine relevant test indicators. The three spraying time points (EL34, EL35, EL38) corresponded to three key developmental stages of Kyoho grapes: early veraison, mid-veraison, and pre-maturation. During this phase, antioxidant synthesis in the fruit is active, and storability gradually forms, making it an optimal window for exogenous treatments (Li et al., 2020; Yu et al., 2023).

### 2.2 Assay method

#### 2.2.1 Determination of grape fruit quality indicators

##### 2.2.1.1 Total soluble solids and titratable acidity of grape berries

The determinations of total soluble solids (TSS) were performed using a Luheng Biology LH-T55 digital refractometer, with each treatment repeated 10 times.

The contents of titratable acid (TA) were determinated as follow, 10 grapes were taken from each treatment and weighed. After juicing the berries, the juice was filtered through gauze, and the volume of the obtained juice was measured. 10 mL of the juice was taken, and 4 drops of 1% phenolphthalein reagent were added. The mixture was titrated with 0.1 mmol·L^-1^ NaOH solution, with each treatment repeated 5 times.

##### 2.2.1.2 Antioxidant content of berries

The contents of total phenols, flavonoids, anthocyanins, and ascorbic acid in grape berries were determined with reference to the method by Cao Jiankang (Cao et al., 2019).

Determination of anthocyanins, total phenols, and flavonoids: The hydrochloric acid-methanol method was used with slight modifications. For each treatment group, 3 grams of mixed peel samples from 30 berries were ground into powder in liquid nitrogen. 0.1 grams of the powder was dissolved in 1% hydrochloric acid-methanol solution and diluted to 10 mL. After vortex mixing, the mixture was shaken for 24 hours at 4°C in the dark for extraction (Ji et al., 2018). Although the extraction time is long, the low temperature and dark conditions minimize the degradation of phenolic compounds, recovery verification was not conducted in this study; this is inferred from the reference. The remaining steps followed the original method, with 9 replicates set for each treatment.

Determination of ascorbic acid content: The bathophenanthroline (BP) method was used to determine the ascorbic acid content in berries, with each treatment repeated 9 times.

#### 2.2.2 Determination of storability index of grape berry

##### 2.2.2.1 Fruit puncture index

A Shimadzu EZ-SX fruit and vegetable quality analyzer was used for the puncture test following the manufacturer’s protocol, with 10 replicates per treatment. Test parameters: A 2 mm probe was used, with a downward speed of 20.00 mm/min (displacement: 10 mm) during the penetration phase and an upward speed of 500.00 mm/min (displacement: 15 mm) during the rebound phase. The “puncture force peak” output by the instrument corresponds to peel puncture strength; the “rebound slope of the displacement-force curve” reflects flesh elasticity, and the “force decay rate after the peak” reflects brittleness. These correlations are based on the instrument operation manual and conventional interpretation of fruit mechanical properties. This test was used to evaluate the effects of each treatment on peel puncture strength, peel elasticity, average flesh firmness, and brittleness ratio.

##### 2.2.2.2 Statistics on berry drop rate and berry rot rate

Rotten and damaged berries were removed before harvesting. During post-harvest storage (0–96 h), the weights of the entire cluster, dropped berries, and rotten berries of each grape cluster were measured separately. Pinching the end of the grape stems, the grape cluster placed flat on the laboratory bench was lifted gently; those that fell onto the bench were regarded as dropped berries, and berries with broken and rotten peels on the cluster and the bench were regarded as rotten berries.


$$\text{Berry drop rate}(\%)=\frac{Falling\;grain\;quality}{Whole\;spike\;fruit\;quality}\times 100\text{\% }$$

$$\text{Rotten fruit rate}(\%)=\frac{Rotten\;fruit\;quality}{\text{Whole spike fruit quality}}\times 100\%$$

##### 2.2.2.3 Degree of browning of grape stems

Weigh 1 g of grape stems sample, cut it into pieces, quickly grind it thoroughly in liquid nitrogen, add distilled water to make the volume up to 10.0 mL, and let it stand for extraction for 2 h. Add 5.0 mL of the extract to each of three 10 mL centrifuge tubes, then add 5.0 mL of 95% ethanol. After vortexing to mix well, centrifuge at 5400 rpm for 10 min. Take the supernatant to determine the absorbance value at A420, using a mixture of 5 mL distilled water and 95% ethanol as the reference. The browning degree is expressed as OD420/g (fresh weight).

##### 2.2.2.4 Grape pedicel tensile resistance and fruit brush length and thickness

Using a Shimadzu EZ-SX fruit and vegetable quality tester, with each treatment repeated 10 times, the tensile tests of grape pedicel were conducted using the method attached to the instrument: the grape pedicel was secured with a fruit and vegetable clamp probe and a metal wire for the tensile test. The probe’s upward speed during the test was 30.00 mm/min (displacement: 15 mm), and the effects of each treatment on maximum tensile strength, tensile deformation, and tensile energy were recorded.

The length and thickness of the fruit brush pulled down from the grape pedicel test were measured using an Ezerui ARZ-1332 digital vernier caliper, the fruit brush refers to the point where the grape pedicel connects to the berry, with the same 10 repetitions.

##### 2.2.2.5 Lignin content of grape berries

The procedure was adapted from the method described by Wang with minor modifications (Wang et al., 2007). Briefly, 1 g of pulp tissue was homogenized in 4 mL of pre-chilled 95% ethanol. The homogenate was centrifuged at 9,000 rpm for 10 min at 4°C, and the supernatant was discarded. Then, 4.5 mL of an ethanol–heptane mixture was added, vortexed thoroughly, and centrifuged again at 9,000 rpm for 2 min at 4°C; this step was repeated three times. After discarding the supernatant, 4.5 mL of 95% ethanol was added, vortexed, and centrifuged at 6,000 rpm for 2 min at 4°C; this step was also repeated three times. Subsequently, 1 mL of 2 mol·L^-1^ NaOH solution was added and vortexed thoroughly. To the resulting mixture, 2 mL of glacial acetic acid and 0.1 mL of 7.5 mol·L^-1^ hydroxylamine hydrochloride solution were sequentially added. After thorough vortex mixing, the sample was centrifuged at 9,000 rpm for 5 min at 4°C. The supernatant was collected and adjusted to a final volume of 10 mL with acetic acid solution. Absorbance was measured at 280 nm using a UV spectrophotometer, expressed as absorbance per unit fresh weight (OD280/g fresh weight). The 280 nm UV spectrophotometric method has limited specificity and may be interfered with by other aromatic compounds; subsequent results will be comprehensively interpreted in conjunction with enzyme activity and gene expression data related to the phenylpropane metabolic pathway. Due to experimental constraints, the more specific thioglycolic acid method was not employed, which represents a limitation of this study.

##### 2.2.2.6 Detection and identification of fungal flora on the surface of the peel

Isolation, purification, and identification of fungi: With reference to the method by Li Xue (Li X et al., 2016). with slight modifications, potato sucrose agar (PSA) medium was used for the isolation and purification culture of fungi. Five grape berries from each treatment were randomly selected for tissue isolation. Each berry was soaked in 75% alcohol for 30 seconds, then 3 pieces of epidermal tissue (5×5 mm) with a depth of 1–2 mm were cut using a scalpel. The epidermal tissue was placed on the medium with the epidermal side down and incubated in a constant temperature incubator at 28°C. Marking was started when colonies began to form; after 5 days, when the colonies stabilized, the number of colonies was counted and numbered. Subsequently, the colonies were transferred to new PSA medium for purification culture. In the process of identifying fungal species, visual identification was conducted based on characteristics such as colony morphology, color, and size, and the number of colonies and the bacterial carrying rate were counted. Preliminary identification based on colony morphology is subjective and was corrected using ITS sequencing data. The lack of molecular identification at all time points is a limitation of this study.

The statistics of isolated colonies referred to the number of fungal colonies obtained through isolation and culture (Li X et al., 2016).

The fungal contamination rate refers to the ratio (%) of the number of fungal tissue blocks to the total number of tissue blocks, reflecting the amount of fungi on the surface of the fruit, and can be used to compare the effects of different treatments on fungi (Liu, 2022).

ITS detection and identification: The experimental design focused on changes in the fungal community at the beginning and end of storage, berries stored for 0 hours and 96 hours under different treatments were selected. For each treatment, the skins of thirty berries were sampled and divided into three parts, and sent to Shanghai Maji Biomedical Technology Co., Ltd. for ITS sequencing to analyze peel surface fungal diversity. The specific workflow was as follows: Total DNA was extracted from peel samples using the CTAB method. The ITS2 region was amplified with primers ITS3 (5’-GCATCGATGAAGAACGCAGC-3’) and ITS4 (5’-TCCTCCGCTTATTGATATGC-3’). Sequencing data were quality-controlled using Trimmomatic (parameters: Leading=3, Trailing=3, SlidingWindow=4:15, MinLen=36), OTUs were clustered using USEARCH (97% similarity), and taxonomic annotation was performed via BLAST against the UNITE database (v8.2).

#### 2.2.3 Correlation enzyme activity assays

##### 2.2.3.1 Cellulase and pectinase activity assays

With reference to the method by Cao Jiankang (Cao et al., 2019), the activities of cellulase and pectinase were determined using the galacturonic acid colorimetric method and sodium methylcellulose colorimetric method, respectively.

##### 2.2.3.2 Oxidase and antioxidant enzyme activity assays

The following enzyme activity indicators were all determined using corresponding kits produced by Beijing Solarbio Science & Technology Co., Ltd.

PPO: Detected using the Polyphenol Oxidase (PPO) Activity Assay Kit (catalog number: BC0190), and the kit method is based on the catechol method.

POD: Detected using the Peroxidase (POD) Activity Assay Kit (catalog number: BC0090).

SOD: Detected using the Superoxide Dismutase (SOD) Activity Assay Kit (catalog number: BC5160), and the kit method is based on the WST-1 method.

##### 2.2.3.3 Assay of enzyme activity associated with phenylpropane metabolic pathway

PAL: Detected using the Phenylalanine Ammonia-Lyase (PAL) Activity Assay Kit (catalog number: BC0210), and the kit method is based on the trans-cinnamic acid colorimetric method.

C4H: Detected using the Cinnamate-4-Hydroxylase (C4H) Activity Assay Kit (catalog number: BC4080). The kit method is based on the NADPH colorimetric method.

4CL: Detected using the 4-Coumarate: Coenzyme A Ligase (4CL) Activity Assay Kit (catalog number: BC4220). The kit method is based on the 4-coumaroyl-CoA colorimetric method.

#### 2.2.4 Determination of gene expression levels related to phenylpropanoid metabolic pathway

Peel samples were ground into powder in liquid nitrogen. Total RNA was extracted using a Plant Total RNA Extraction Kit (Catalog No.: Cat5101050, Xinjing), and cDNA was synthesized by reverse transcription with total RNA as the template using the Takara PrimeScript™ RT-PCR Kit. Key enzyme genes involved in the anthocyanin and lignin metabolic pathways were selected for quantitative real-time PCR (qPCR) analysis, including *VvPA*L (XM_006723833.5), *Vv4CL* (XM_002274958.5), *VvCHS* (NM_001281135.1), *VvANS* (NM_001280950.1), *VvCCR* (XM_034821066.1), *VvCAD* (XM_002279682.3), and *VvCOMT* (XM_010662020.3). *VvACTIN* (XM_002273532) was used as the reference gene, which is widely applied in grape gene expression studies (Lai et al., 2019; Su et al., 2021). Due to experimental constraints, a second reference gene was not included, and data stability was ensured through technical replicates (3 times).

qPCR primers were designed using the NCBI primer design tool following qPCR primer design principles, and primer information is shown in **Table 2**. All qPCR primers were verified by melting curve analysis, showing a single peak with no non-specific amplification, and the amplification efficiency ranged from 96% to 103%. A 10 μL qPCR amplification system was used: 5 μL of 2×SuperReal PreMix Plus, 0.6 μL of forward and reverse primer mixture, 1 μL of cDNA template, and 3.2 μL of ddH_2_O. The reaction conditions consisted of seven stages: 95°C for 10 min, 95°C for 10 s, 59°C for 10 s, 72°C for 20 s, 95°C for 15 s, 60°C for 1 min, and 95°C for 1 s. The heating and cooling rates for the first six stages were all 1.6°C/s, and the heating rate for the last stage was 0.15°C/s, with a total of 40 cycles. Finally, melting curve determination was performed. qPCR results were analyzed using the relative quantification method (2^-ΔΔt^ method), with 3 technical replicates per treatment.

**Table 2:** Primer information.

Gene name	Forward primer 5’-3’	Reverse primer 5’-3’	Product length	Amplification efficiency
*VvACTIN*	CGCAAATGTTATGCACGCCT	CTTCTTCTTTGGCCTCAACTGG	90	99.3%
*VvPAL*	CAAAGGCACCGACAGCTATG	ATCCCAGCATTCAAGAACCTGAT	114	98.2%
*Vv4CL*	AAAGGCTTCCAGGTACCGC	GCAACAGGAACTTCACCTGC	116	101.5%
*VvCHS*	AGTTCAAGCGCATGTGTGAT	GCCATGTAGGCACAGACGTT	100	97.8%
*VvANS*	CCAAGCGACTACGTTCCAGC	CTTCCAATCCCAACCCAAGC	100	100.3%
*VvCCR*	TACGTTGCTTCATGGGTCGT	TCTACCTGCAAAAGCATGGGT	89	96.7%
*VvCAD*	CTCTGGTCTTGGGGCGGAAG	TTGGCACAGAACTCCAGCAT	85	102.8%
*VvCOMT*	GCAACGGCAGTGTCCTCAAT	AACATGCTCAATGCCAGGGT	116	103.0%

#### 2.2.5 Comprehensive evaluation of entropy weight TOPSIS

Entropy-weighted TOPSIS comprehensive evaluation analysis was performed on the fruit quality and storability indicators at 5 storage times, respectively. Among them, TA content, berry drop rate, rotten berry rate, and browning degree were defined as negative indicators (within a certain range, the smaller the value, the better the treatment effect). Before the analysis, the negative indicators were first converted to positive ones, and then the converted positive indicators and the remaining indicators were subjected to interval transformation (default interval 1 - 2) to eliminate the impact caused by different units of each indicator, i.e., dimension processing. Then, the processed data were analyzed by entropy-weighted TOPSIS comprehensive evaluation using the SPSSAU online tool. Entropy-weighted TOPSIS is a combination of the entropy weight method (entropy value method) and the TOPSIS method, and its calculation is divided into two steps. First: The first step of entropy-weighted TOPSIS is to calculate weight values using the entropy weight method, and weight the data to obtain new data (automatically completed by the algorithm); the weight values of each evaluation indicator item are obtained, and then the data are weighted by the weight values for the TOPSIS analysis in the second step. Second: The second step is to apply the TOPSIS method using the new data to finally complete the analysis.

Compared with principal component analysis (PCA), this method can objectively assign weights to indicators through the entropy value method, avoiding subjective weighting bias. It can also quantify the closeness of samples to the optimal/worst solutions, making it more suitable for multi-indicator (quality + storability) evaluation.

### 2.3 Data analysis

Data were organized using WPS Office, charts were generated using GraphPad Prism 9.5, One-way analysis of variance (ANOVA) was performed using SPSS 26 software, followed by Duncan’s new multiple range test for *post-hoc* analysis (P ≤ 0.05). Normality and homogeneity of variance assumptions were verified. All experimental data are presented as mean ± standard deviation. Entropy weight TOPSIS comprehensive evaluation analysis was conducted using the SPSSAU online tool.

## 3 Results and analysis

### 3.1 Sugars and acids

As shown in **Table 3**, during the 0–96 h storage period, the TSS content of all cCA and PC treatment groups (T1 - T6) was significantly higher than that of the control group (CK). Among them, the T6 treatment exhibited the most prominent effect, with a 6.88% - 14.67% increase compared to CK. The TA content of most treatment groups decreased with the extension of storage time, and there were significant differences between treatments: in the early storage stage (0–48 h), the T3 treatment had the lowest TA content, which was 25.40% - 36.82% lower than that of CK; in the late storage stage (48–96 h), the T6 treatment had the lowest TA content, which was 13.74% - 31.83% lower than that of CK. The solid-acid ratio generally increased with the extension of storage time: at 24 h, the T3 treatment had the highest solid-acid ratio, which was 49.12% higher than that of CK; at 0, 48, 72, and 96 h, T6 treatment had the highest ratio, increasing by 79.09%, 56.67%, 42.10%, and 28.64% compared to CK, respectively. The above results indicate that CA and its potassium salt (PC) treatments can improve the sugar-acid balance of fruits, and high-concentration treatments (T3, T6) show stage-specific advantages.

**Table 3:** Comparison of soluble solids, titratable acids and solid-acid ratios for different treatments.

Storage time(d)	Treatments	Soluble solids (%)	Titratable acid (%)	Solid-acid ratio
0 h	CK	16.60 ± 0.26d	0.35 ± 0.00a	47.15 ± 0.58f
	T0	16.50 ± 0.10d	0.28 ± 0.00e	58.19 ± 0.48d
	T1	17.23 ± 0.15c	0.33 ± 0.00c	52.99 ± 0.49e
	T2	17.30 ± 0.10c	0.34 ± 0.00b	51.38 ± 0.15e
	T3	17.80 ± 0.10b	0.22 ± 0.00g	80.04 ± 1.68b
	T4	16.67 ± 0.12d	0.24 ± 0.00f	69.08 ± 0.94c
	T5	17.07 ± 0.12c	0.30 ± 0.01d	57.14 ± 1.09d
	T6	19.03 ± 0.06a	0.23 ± 0.00g	84.45 ± 1.82a
24 h	CK	16.93 ± 0.12d	0.22 ± 0.01b	78.22 ± 4.17d
	T0	16.97 ± 0.21d	0.20 ± 0.01c	82.86 ± 3.07c
	T1	17.17 ± 0.06d	0.23 ± 0.01a	74.86 ± 3.10d
	T2	18.03 ± 0.06c	0.21 ± 0.00bc	86.49 ± 1.22c
	T3	18.87 ± 0.21b	0.16 ± 0.00e	116.64 ± 2.20a
	T4	17.07 ± 0.12d	0.22 ± 0.00b	78.55 ± 1.66d
	T5	17.93 ± 0.06c	0.21 ± 0.00bc	84.95 ± 0.81c
	T6	19.10 ± 0.10a	0.19 ± 0.00d	98.50 ± 0.43b
48 h	CK	17.43 ± 0.06e	0.20 ± 0.01a	86.49 ± 3.21g
	T0	17.23 ± 0.06f	0.18 ± 0.00b	94.54 ± 0.63f
	T1	17.80 ± 0.17d	0.17 ± 0.00d	104.40 ± 1.00e
	T2	18.00 ± 0.10bc	0.15 ± 0.00f	123.75 ± 1.46c
	T3	18.07 ± 0.12b	0.14 ± 0.00fg	126.77 ± 2.33b
	T4	17.83 ± 0.12cd	0.16 ± 0.00e	109.49 ± 0.65d
	T5	18.13 ± 0.06b	0.18 ± 0.00c	102.81 ± 1.66e
	T6	18.63 ± 0.06a	0.14 ± 0.00g	135.50 ± 1.14a
72 h	CK	16.87 ± 0.06f	0.17 ± 0.00bc	97.28 ± 0.71e
	T0	17.13 ± 0.06e	0.19 ± 0.00a	89.11 ± 1.50f
	T1	18.33 ± 0.06c	0.18 ± 0.00b	101.69 ± 0.76de
	T2	18.67 ± 0.06b	0.17 ± 0.00bc	107.56 ± 3.05cd
	T3	18.90 ± 0.10a	0.15 ± 0.00e	128.94 ± 1.43b
	T4	17.83 ± 0.06d	0.16 ± 0.00d	110.04 ± 3.05c
	T5	17.93 ± 0.06d	0.17 ± 0.00c	104.47 ± 0.84cd
	T6	18.90 ± 0.10a	0.14 ± 0.01f	138.23 ± 9.60a
96 h	CK	17.07 ± 0.06f	0.16 ± 0.00c	104.24 ± 1.35d
	T0	17.53 ± 0.06e	0.18 ± 0.00a	95.50 ± 0.91e
	T1	18.40 ± 0.10c	0.18 ± 0.00a	102.76 ± 1.80d
	T2	18.50 ± 0.10bc	0.17 ± 0.00b	109.22 ± 1.82c
	T3	18.67 ± 0.12b	0.15 ± 0.00d	123.36 ± 2.71b
	T4	17.90 ± 0.10d	0.16 ± 0.00c	110.45 ± 3.38c
	T5	18.50 ± 0.10bc	0.15 ± 0.00e	126.19 ± 0.58b
	T6	18.93 ± 0.12a	0.14 ± 0.00f	134.09 ± 1.05a

The data in the table are presented as mean ± standard deviation. Different lowercase letters indicate significant differences between treatments at the same storage time, P ≤ 0.05. The same applies below.

### 3.2 Antioxidant substances

**Figure 1:**
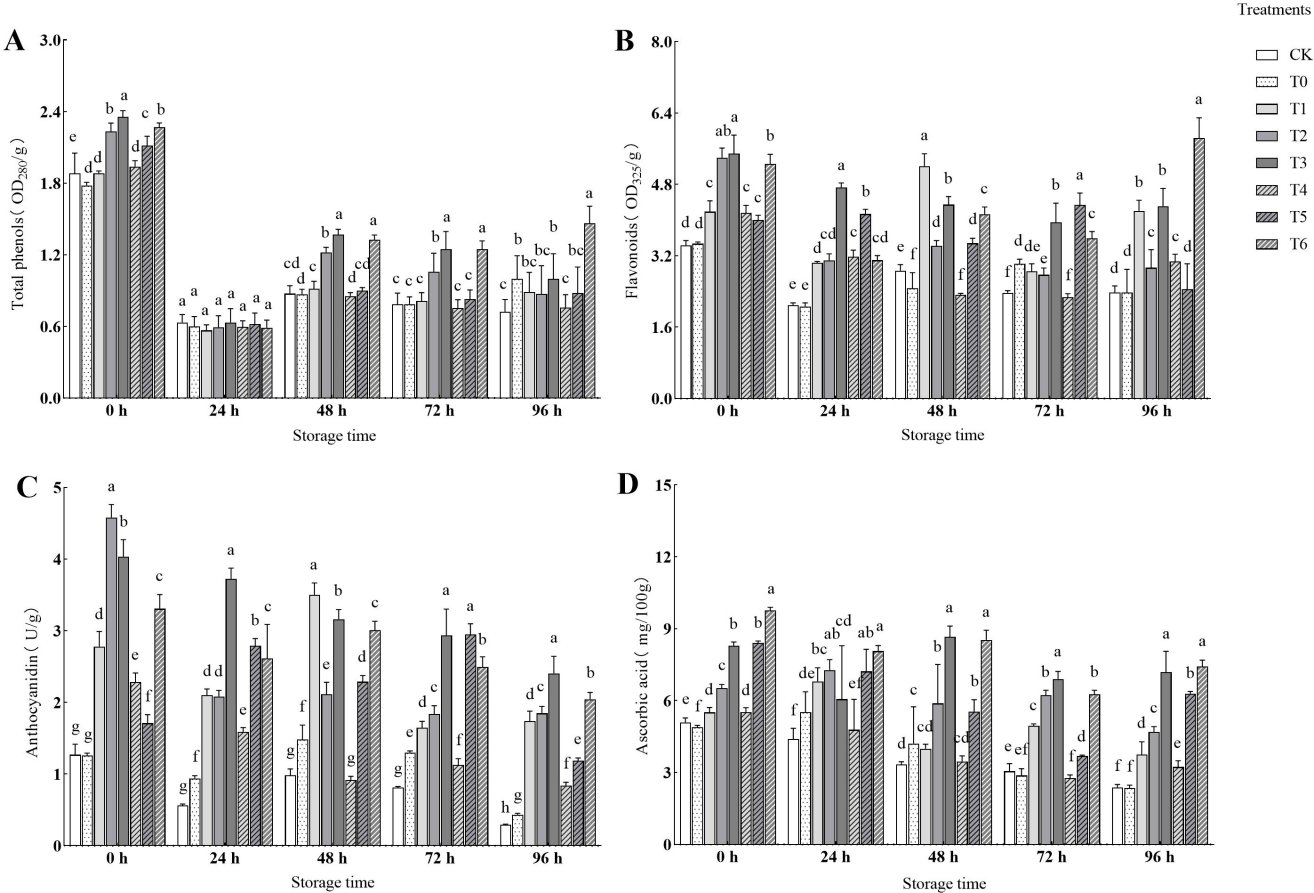
Effects of experimental treatment on the contents of total phenols, flavonoids, anthocyanins and ascorbic acid in berries. The data in the figure are presented as mean ± standard deviation. **(A)** Effects of different treatments on the total phenol content in fruits. **(B)** Effects of different treatments on the flavonoid content in fruits. **(C)** Effects of different treatments on the anthocyanin content in fruits. **(D)** Effects of different treatments on the ascorbic acid content in fruits. Different lowercase letters indicate significant differences between treatments at the same storage time, P ≤ 0.05. The same applies below.

As shown in **Figure 1**, CA and PC treatments significantly regulated the contents of total phenols, flavonoids, anthocyanins, and ascorbic acid in fruits, and most indicators increased with the increase of treatment concentration. The total phenol content was the highest in the T3 treatment at 48–72 h (56.70% - 59.08% higher than CK) and in T6 treatment at 96 h (102.68% higher than CK). The contents of flavonoids and ascorbic acid both reached their peaks at 96 h, and the T6 treatment was the best, increasing by 145.89% and 212.18% compared to CK, respectively. The variation pattern of anthocyanin content was more complex, but T3 treatment had the highest content at 96 h (723.27% higher than CK). In general, high-concentration treatments (T3, T6) can effectively enhance the antioxidant capacity of grape berries, among which T6 treatment shows the most stable effect at the late storage stage (96 h).

### 3.3 Fruit puncture and pedicel tensile mechanical properties

As shown in **Tables 4** and **5**, compared with CK, T3 and T6 treatments significantly maintained the peel puncture strength, peel elasticity, flesh firmness, and brittleness ratio during storage. At the late storage stage (96 h) with practical application significance: T3 treatment had the highest peel puncture strength and tensile deformation, with the peel puncture strength increasing by 75.68% compared to CK and the tensile deformation increasing by 123.31% compared to CK; T6 treatment had the highest flesh firmness and maximum tensile strength, with the flesh firmness increasing by 156.28% compared to CK and the maximum tensile strength increasing by 173.24% compared to CK. These two treatments are the optimal choices for maintaining fruit mechanical properties. Among them, T6 treatment performs prominently in terms of firmness and tensile strength, while T3 treatment has more advantages in terms of peel strength and deformation capacity.

**Table 4:** Comparison of puncture indexes of fruits under different treatments.

Storage time (d)	Treatments	Peel puncture strength(N)	The elasticity of the peel (mm)	Average flesh hardness(N)	Brittleness ratio (%)
0 h	CK	1.90 ± 0.49c	3.02 ± 0.66b	0.24 ± 0.02b	9.66 ± 3.31b
	T0	2.14 ± 0.65bc	3.12 ± 0.70b	0.28 ± 0.09ab	13.17 ± 3.10b
	T1	2.24 ± 0.47bc	3.21 ± 0.62b	0.24 ± 0.01b	12.13 ± 2.96b
	T2	2.38 ± 0.61bc	3.49 ± 0.70b	0.33 ± 0.16ab	14.76 ± 3.14b
	T3	2.67 ± 0.79ab	4.33 ± 0.35a	0.48 ± 0.32a	21.99 ± 10.39a
	T4	2.19 ± 0.56bc	3.36 ± 0.63b	0.25 ± 0.03b	13.49 ± 4.87b
	T5	2.43 ± 0.42bc	3.40 ± 0.55b	0.29 ± 0.04ab	15.51 ± 5.78ab
	T6	3.25 ± 0.27a	3.87 ± 0.87ab	0.46 ± 0.22a	16.75 ± 4.75ab
24 h	CK	1.97 ± 0.15c	2.94 ± 0.16d	0.24 ± 0.07b	9.82 ± 3.51a
	T0	2.27 ± 0.48c	3.13 ± 0.43cd	0.24 ± 0.03b	10.48 ± 3.36a
	T1	2.20 ± 0.32c	3.14 ± 0.48cd	0.26 ± 0.05b	13.20 ± 2.14a
	T2	2.61 ± 0.39bc	3.81 ± 0.78bc	0.31 ± 0.11b	13.88 ± 4.98a
	T3	3.20 ± 0.21ab	4.60 ± 0.59a	0.52 ± 0.27a	14.66 ± 6.91a
	T4	2.44 ± 0.47c	3.41 ± 0.68bcd	0.25 ± 0.07b	10.59 ± 3.46a
	T5	2.49 ± 0.62c	3.85 ± 0.58b	0.30 ± 0.07b	14.12 ± 4.56a
	T6	3.60 ± 0.97a	4.07 ± 0.37ab	0.33 ± 0.04b	15.03 ± 3.22a
48 h	CK	2.35 ± 0.71b	3.23 ± 0.31c	0.23 ± 0.04b	10.66 ± 2.15b
	T0	2.08 ± 0.17b	3.51 ± 0.60bc	0.24 ± 0.03b	12.07 ± 1.02b
	T1	2.12 ± 0.37b	3.16 ± 0.14c	0.22 ± 0.02b	9.01 ± 1.80b
	T2	2.55 ± 0.33ab	3.60 ± 0.68abc	0.25 ± 0.13b	12.66 ± 2.57b
	T3	2.78 ± 0.61ab	4.08 ± 0.31ab	0.27 ± 0.04b	13.17 ± 3.42b
	T4	2.15 ± 0.34b	3.53 ± 0.60bc	0.25 ± 0.02b	12.02 ± 2.45b
	T5	2.35 ± 0.22b	4.05 ± 0.72ab	0.28 ± 0.05b	12.17 ± 1.62b
	T6	3.06 ± 1.01a	4.28 ± 0.72a	0.52 ± 0.36a	17.66 ± 7.12a
72 h	CK	1.91 ± 0.17bc	3.35 ± 0.28c	0.28 ± 0.15d	10.07 ± 2.15de
	T0	1.43 ± 0.19c	2.88 ± 0.21c	0.43 ± 0.15cd	11.48 ± 5.12c
	T1	2.35 ± 0.39b	4.45 ± 0.47b	0.26 ± 0.07d	6.21 ± 0.77e
	T2	3.27 ± 0.49a	4.48 ± 1.22b	0.57 ± 0.26bc	16.54 ± 3.96abc
	T3	3.56 ± 0.68a	5.44 ± 0.36a	0.76 ± 0.28b	19.07 ± 4.60ab
	T4	3.09 ± 0.55a	4.16 ± 0.35b	0.28 ± 0.13d	12.55 ± 0.85cd
	T5	3.26 ± 0.94a	4.23 ± 1.02b	0.69 ± 0.19b	14.34 ± 3.60bcd
	T6	3.60 ± 0.56a	4.81 ± 0.53ab	1.06 ± 0.30a	20.67 ± 6.34a
96 h	CK	2.10 ± 0.39de	3.29 ± 0.57c	0.22 ± 0.11b	11.05 ± 2.70bc
	T0	2.03 ± 0.48e	4.21 ± 0.31ab	0.35 ± 0.16ab	7.82 ± 0.55c
	T1	2.38 ± 0.47cde	4.22 ± 0.58ab	0.37 ± 0.16ab	7.35 ± 2.48c
	T2	2.98 ± 0.36abc	4.54 ± 1.12ab	0.40 ± 0.21ab	13.05 ± 1.30ab
	T3	3.68 ± 0.48a	4.69 ± 0.68ab	0.51 ± 0.17ab	16.64 ± 4.58a
	T4	2.84 ± 0.78bcd	3.93 ± 0.61bc	0.25 ± 0.04b	10.49 ± 3.47bc
	T5	3.22 ± 0.94ab	4.37 ± 0.74ab	0.44 ± 0.18ab	14.94 ± 1.78ab
	T6	3.07 ± 0.75abc	4.96 ± 0.64a	0.57 ± 0.47a	16.74 ± 8.69a

The data in the table are presented as mean ± standard deviation. Different lowercase letters indicate significant differences between treatments at the same storage time, P ≤ 0.05.

**Table 5:** Comparison of grape stalk tensile indexes under different treatments.

Storage time(d)	Treatments	Maximum tensile strength(N)	Tensile deformation(mm)	Stretch energy (mJ)
0 h	Ck	2.94 ± 0.44b	3.56 ± 0.75a	7.78 ± 1.95b
	T0	3.02 ± 0.45b	3.95 ± 1.32a	7.56 ± 2.30b
	T1	3.55 ± 0.24b	3.96 ± 0.63a	7.81 ± 2.73b
	T2	3.40 ± 0.22b	4.06 ± 0.55a	9.68 ± 1.53b
	T3	3.56 ± 0.43b	4.53 ± 1.16a	10.22 ± 0.85ab
	T4	3.41 ± 0.21b	4.19 ± 0.55a	9.18 ± 2.33b
	T5	3.51 ± 0.47b	4.27 ± 0.44a	9.84 ± 1.54ab
	T6	4.54 ± 0.95a	4.55 ± 0.64a	12.87 ± 4.83a
24 h	Ck	1.88 ± 0.40c	3.05 ± 0.30c	3.32 ± 0.92c
	T0	2.50 ± 0.36bc	2.83 ± 0.47c	5.15 ± 2.42bc
	T1	2.12 ± 0.47bc	3.45 ± 0.48bc	4.13 ± 1.52c
	T2	2.80 ± 0.70b	3.49 ± 0.48bc	5.92 ± 4.68bc
	T3	3.55 ± 0.31a	4.75 ± 1.44a	10.70 ± 1.54a
	T4	2.61 ± 0.57b	3.70 ± 0.50bc	6.03 ± 1.71bc
	T5	3.52 ± 0.65a	4.18 ± 0.73ab	7.61 ± 1.29b
	T6	2.84 ± 0.78b	4.00 ± 0.49ab	6.11 ± 1.73bc
48 h	Ck	0.91 ± 0.53b	1.86 ± 0.21cd	1.03 ± 0.79c
	T0	0.97 ± 0.41b	1.54 ± 0.40d	1.11 ± 0.18c
	T1	1.17 ± 0.45b	1.88 ± 0.18cd	2.96 ± 1.32b
	T2	2.06 ± 0.51a	2.67 ± 0.22ab	1.49 ± 0.76c
	T3	2.63 ± 0.63a	2.74 ± 0.40a	4.73 ± 1.73a
	T4	0.92 ± 0.12b	1.67 ± 0.19d	1.16 ± 0.52c
	T5	2.10 ± 0.63a	2.21 ± 0.57c	3.52 ± 0.98ab
	T6	2.21 ± 0.28a	2.30 ± 0.43bc	3.52 ± 1.72ab
72 h	Ck	0.76 ± 0.48b	1.28 ± 0.24d	1.08 ± 0.47d
	T0	0.63 ± 0.14b	1.67 ± 0.39cd	0.82 ± 0.51d
	T1	0.79 ± 0.48b	1.80 ± 0.42cd	1.35 ± 0.89cd
	T2	1.70 ± 0.56a	2.28 ± 0.30bc	2.39 ± 0.37bc
	T3	2.01 ± 0.37a	2.51 ± 0.54b	3.55 ± 0.33b
	T4	0.52 ± 0.24b	1.19 ± 0.75d	0.49 ± 0.15d
	T5	1.82 ± 0.54a	2.11 ± 0.32bc	3.34 ± 1.64b
	T6	2.21 ± 0.75a	3.36 ± 0.82a	5.25 ± 1.71a
96 h	Ck	0.72 ± 0.45d	1.33 ± 0.63c	0.78 ± 0.48e
	T0	0.48 ± 0.15d	0.81 ± 0.11d	0.22 ± 0.08e
	T1	0.62 ± 0.27d	1.56 ± 0.31c	1.66 ± 0.84cd
	T2	0.99 ± 0.55cd	2.94 ± 0.19a	1.97 ± 1.25c
	T3	1.84 ± 0.26ab	2.97 ± 0.50a	2.91 ± 0.43ab
	T4	0.99 ± 0.41cd	1.65 ± 0.69bc	0.92 ± 0.37de
	T5	1.38 ± 0.71bc	2.11 ± 0.24b	2.34 ± 0.75bc
	T6	1.94 ± 0.38a	2.96 ± 0.46a	3.20 ± 0.64a

The data in the table are presented as mean ± standard deviation. Different lowercase letters indicate significant differences between treatments at the same storage time, P ≤ 0.05.

### 3.4 Berry drop and decay

**Figure 2:**
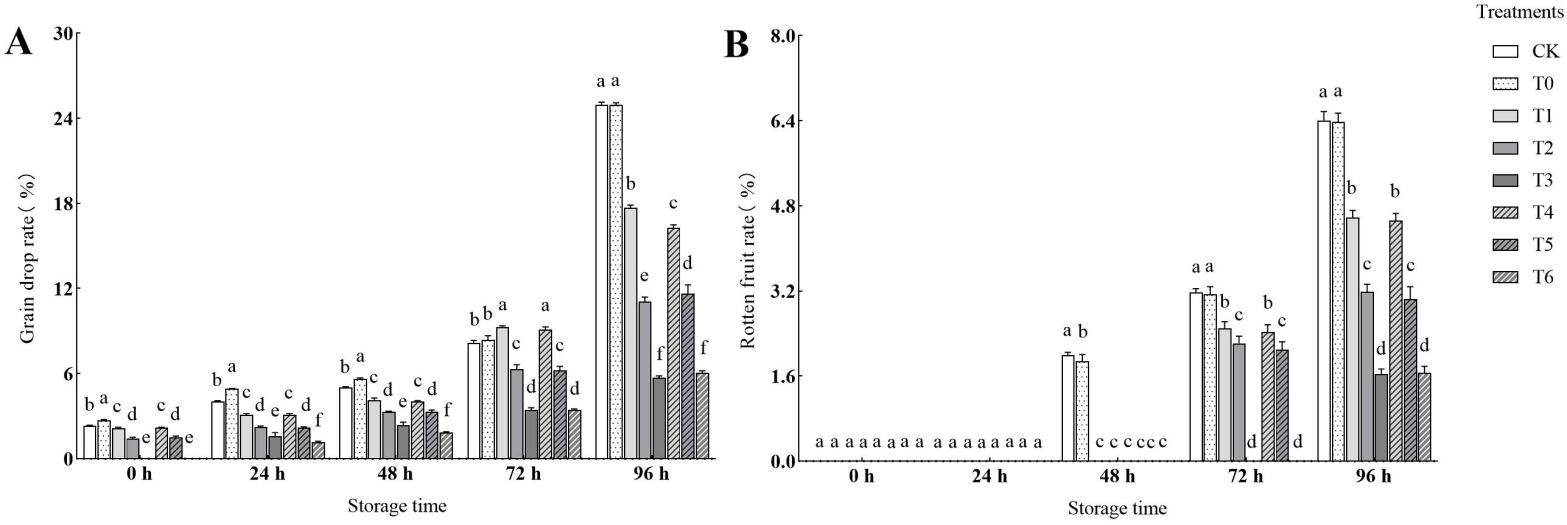
Comparison of berry drop rate and rotten berry rate under different experimental treatments. **(A)** Effects of different treatments on grain drop rate. **(B)** Effects of different treatments on rotten fruit rate.

**Figure 3:**
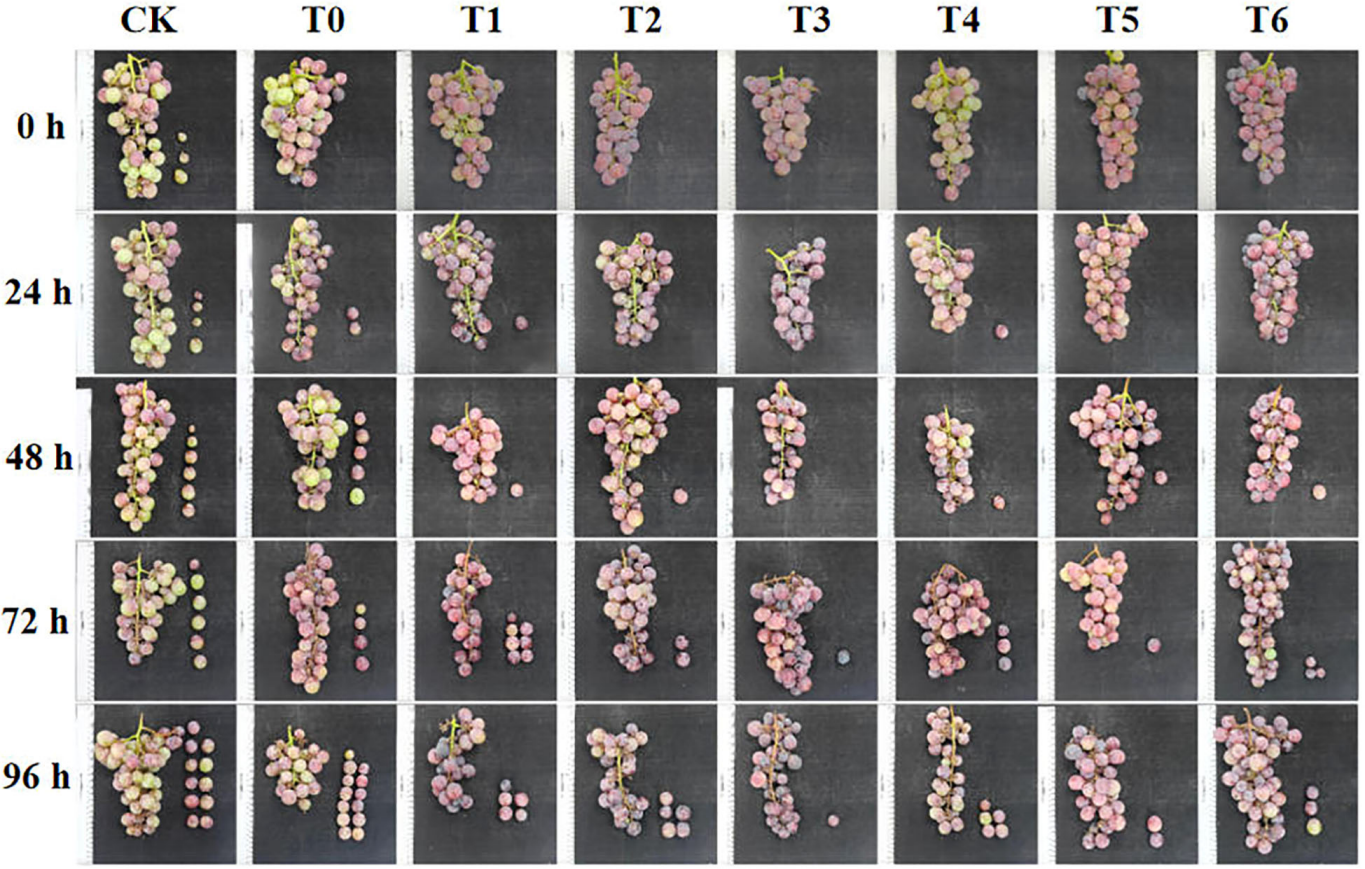
Performance effects of different treatments.

As shown in **Figure 2** and **3**, the fruit drop rate and decay rate increased with the extension of storage time, but all CA/PC treatments (T1 - T6) showed significant inhibitory effects, and the effect increased with the increase of treatment concentration. At 96 h, T3 treatment had the lowest drop rate and decay rate, with the drop rate decreasing by 77.13% compared to CK and the decay rate decreasing by 74.41% compared to CK, followed by T6 treatment. At 72 h, no decayed fruits were found in T3 and T6 treatments, while the CK and T0 treatments began to decay at 48 h. It can be seen that high-concentration treatments (T3, T6) have the strongest ability to reduce fruit loss during storage, and T3 treatment is the optimal choice for controlling late-stage decay and drop.

### 3.5 Characteristics of grape pedicels and lignin content in grape stems

**Figure 4:**
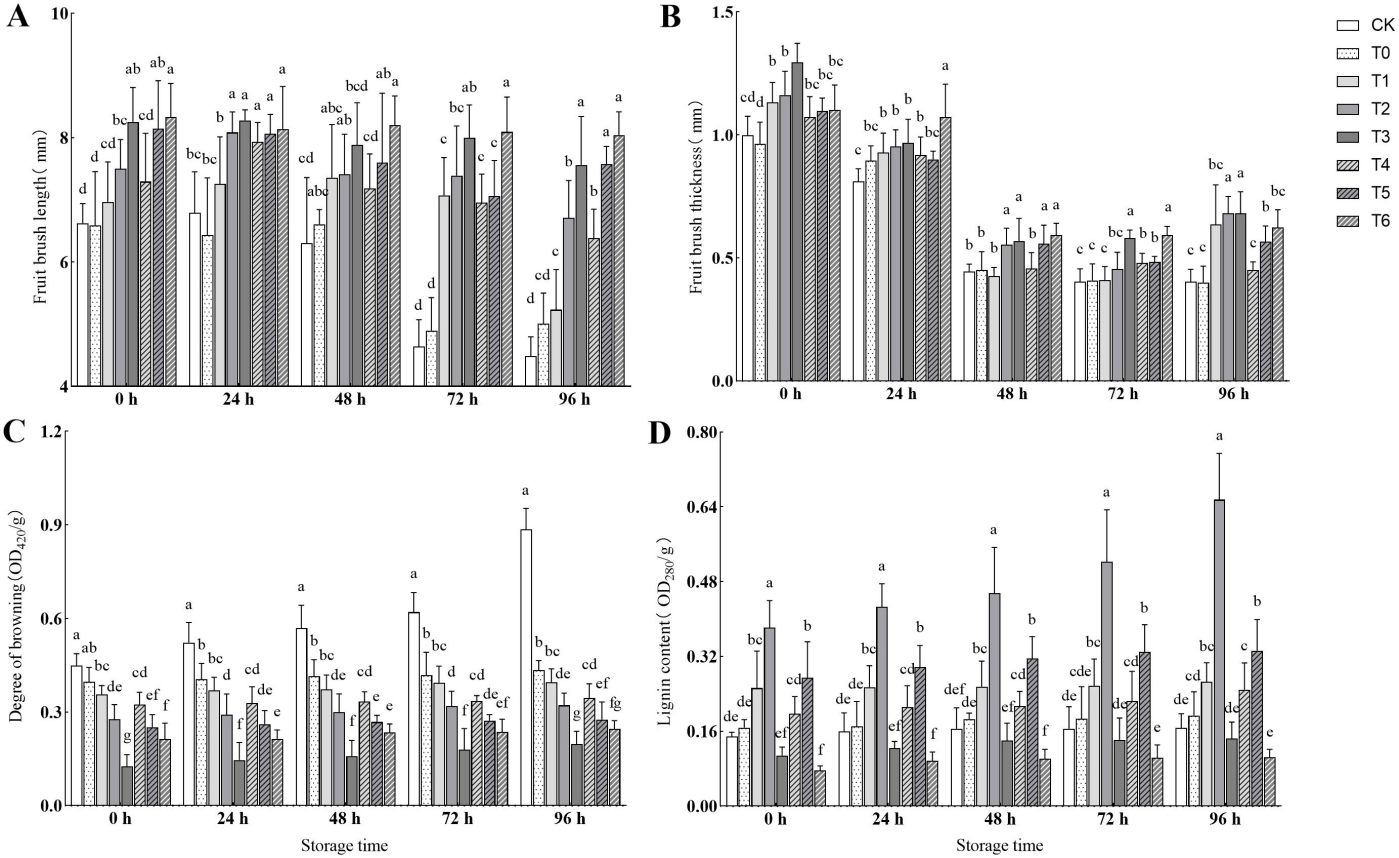
**(A)** Effects of different treatments on fruit brush length. **(B)** Effects of different treatments on fruit brush thickness. **(C)** Effects of different treatments on degree of browning. **(D)** Effects of different treatments on fruit lignin content.

As shown in **Figure 4**, CA and PC treatments significantly regulated the fruit brush length and thickness, grape stems browning degree, and lignin content, and high-concentration treatments generally performed better. In terms of fruit brush length, T6 treatment remained the longest at most time points, increasing by 79.37% compared to CK at 96 h; the fruit brush thickness was alternately led by T3 and T6 treatments, and T3 treatment was the thickest at 96 h, increasing by 69.03% compared to CK; as a key indicator of senescence, the grape stems browning degree was the lowest in T3 treatment at all storage stages, decreasing by 77.75% compared to CK at 96 h. The lignin content showed a trend of “promotion at low concentration and inhibition at high concentration”: T2 treatment significantly increased the lignin content, which was 292.45% higher than that of CK at 96 h, while T6 treatment significantly decreased it, which was 37.36% lower than that of CK at 96 h. This indicates that high-concentration PC can maintain fruit quality by inhibiting excessive lignification.

### 3.6 Surface microbial community

**Figure 5:**
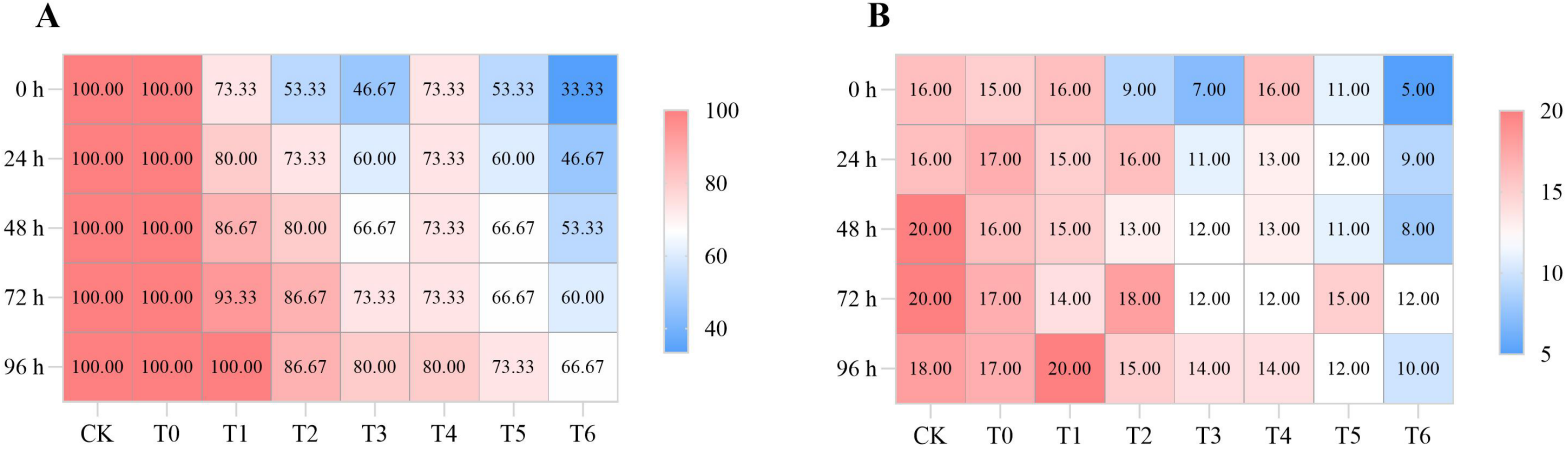
Comparison of carrier rate **(A)** (%) and colony number **(B)** (number) of different test treatments.

**Figure 6:**
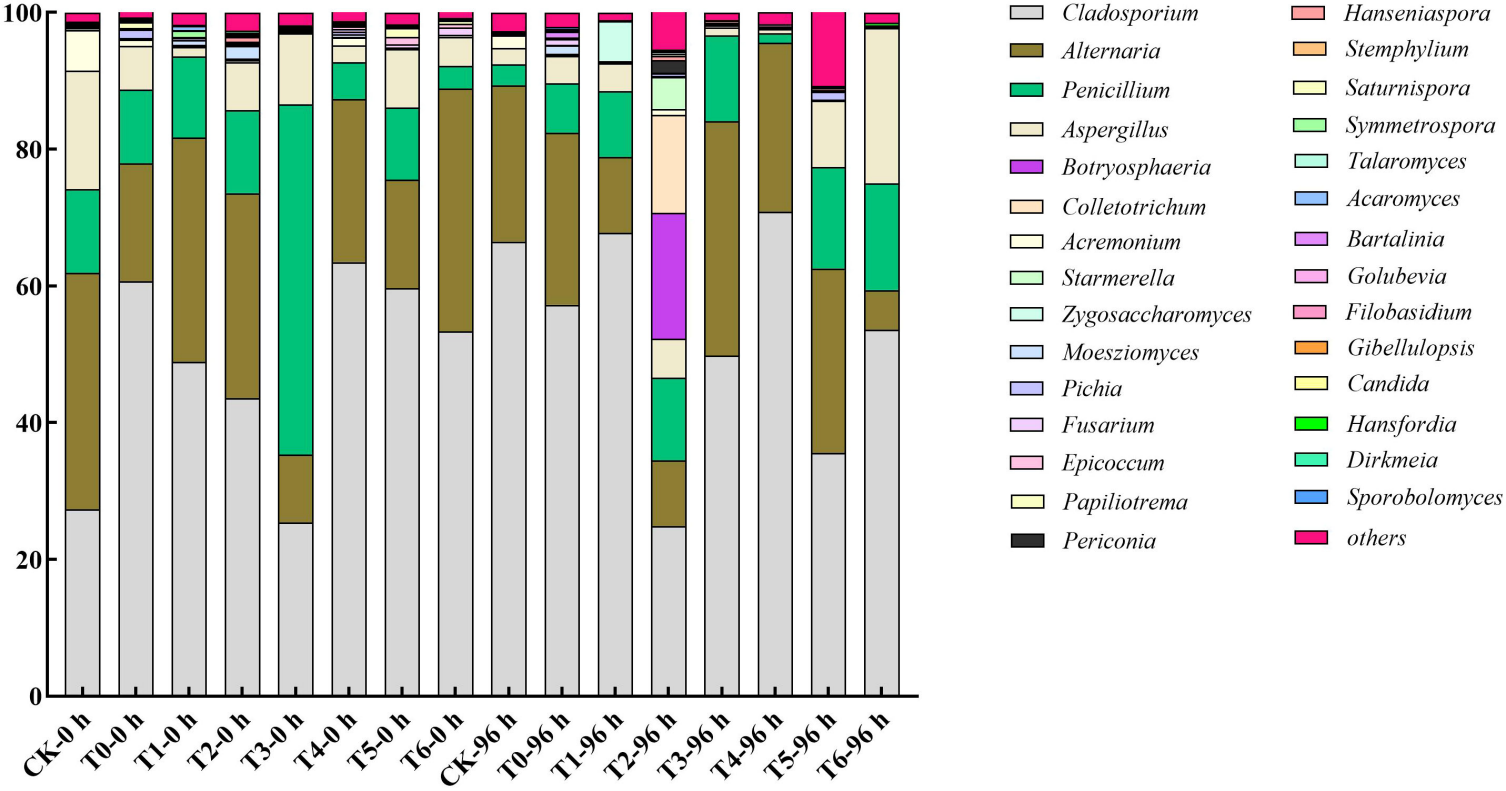
Effects of different treatments on the relative abundance of microflora on grape surface after 0 h and 96 h of storage.

**Figure 7:**
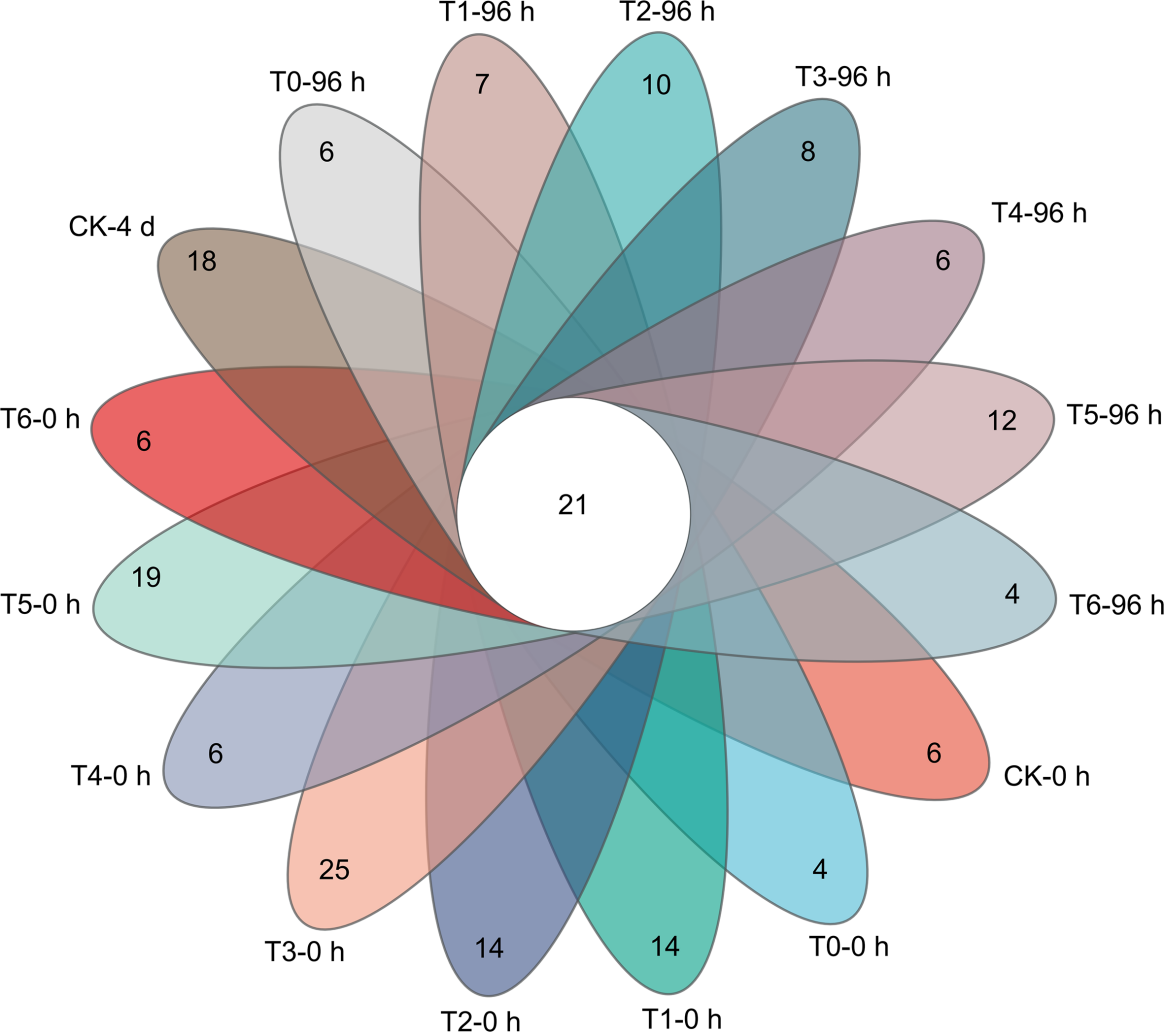
Effects of different treatments on the number of unique and common bacteria on grape surface microbiota.

As shown in **Figure 5**, high-concentration treatments (T3, T6) can effectively inhibit the microbial carrying rate and colony count on the surface of grape berries. Although the inhibitory effect weakens with the extension of time, it is always better than low-concentration treatments. At 96 h, T6 treatment had the lowest colony count, which was 44.44% lower than that of CK, and the carrying rate of T3 treatment was 20.00% lower than that of CK. These two treatments can also change the microbial community structure: T3 treatment effectively inhibits Alternaria, and as shown in **Figures 6** and **7**, T6 treatment stably inhibits Fusarium, and there is a phenomenon of niche replacement of Penicillium by Aspergillus. In terms of long-term microbial control, T6 treatment performs the best, followed by T3 treatment.

### 3.7 Enzyme activities

**Figure 8:**
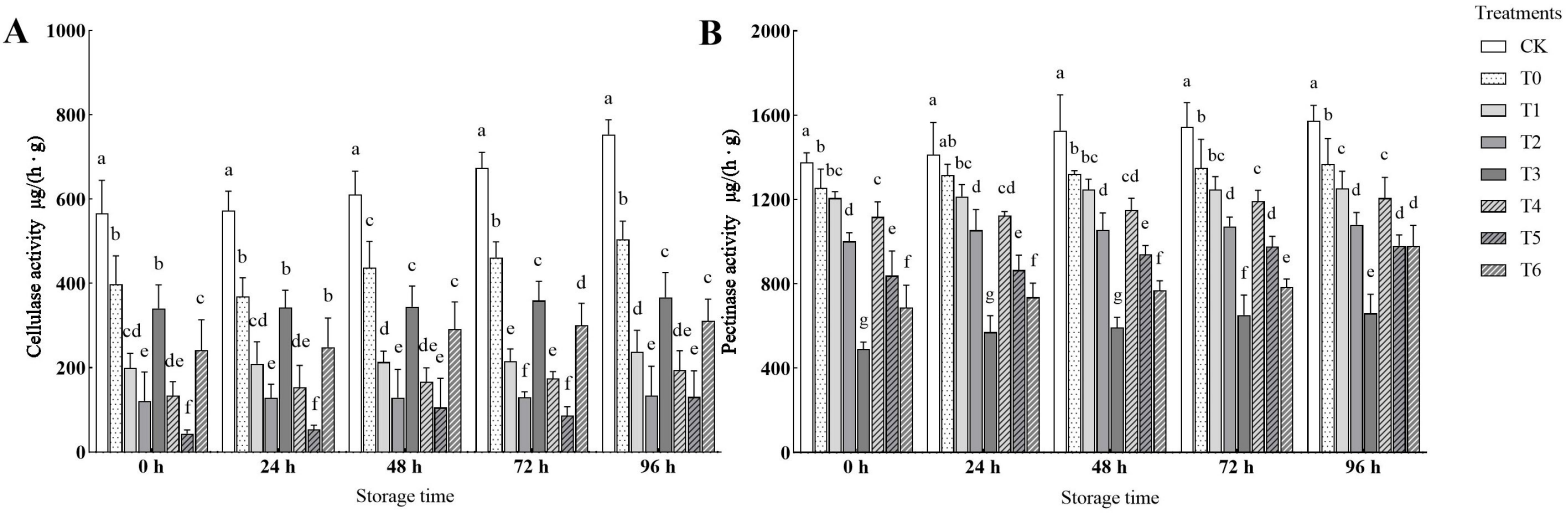
Comparison of the effects of cellulase and pectinase activities in different treatments. **(A)** Effects of different treatments on cellulase activity. **(B)** Effects of different treatments on pectinase activity.

As shown in **Figure 8**, the activities of cellulase and pectinase increased with the extension of storage time, but all treatments (T0 - T6) showed inhibitory effects on them. Among them, T5 treatment had the strongest inhibitory effect on cellulase, which was 82.62% - 92.28% lower than that of CK during storage, and T3 treatment had the best inhibitory effect on pectinase, which was 57.83% - 64.39% lower than that of CK. This indicates that high-concentration CA (T3) and medium-concentration PC (T5) can delay fruit softening by specifically inhibiting the activity of cell wall-degrading enzymes.

**Figure 9:**
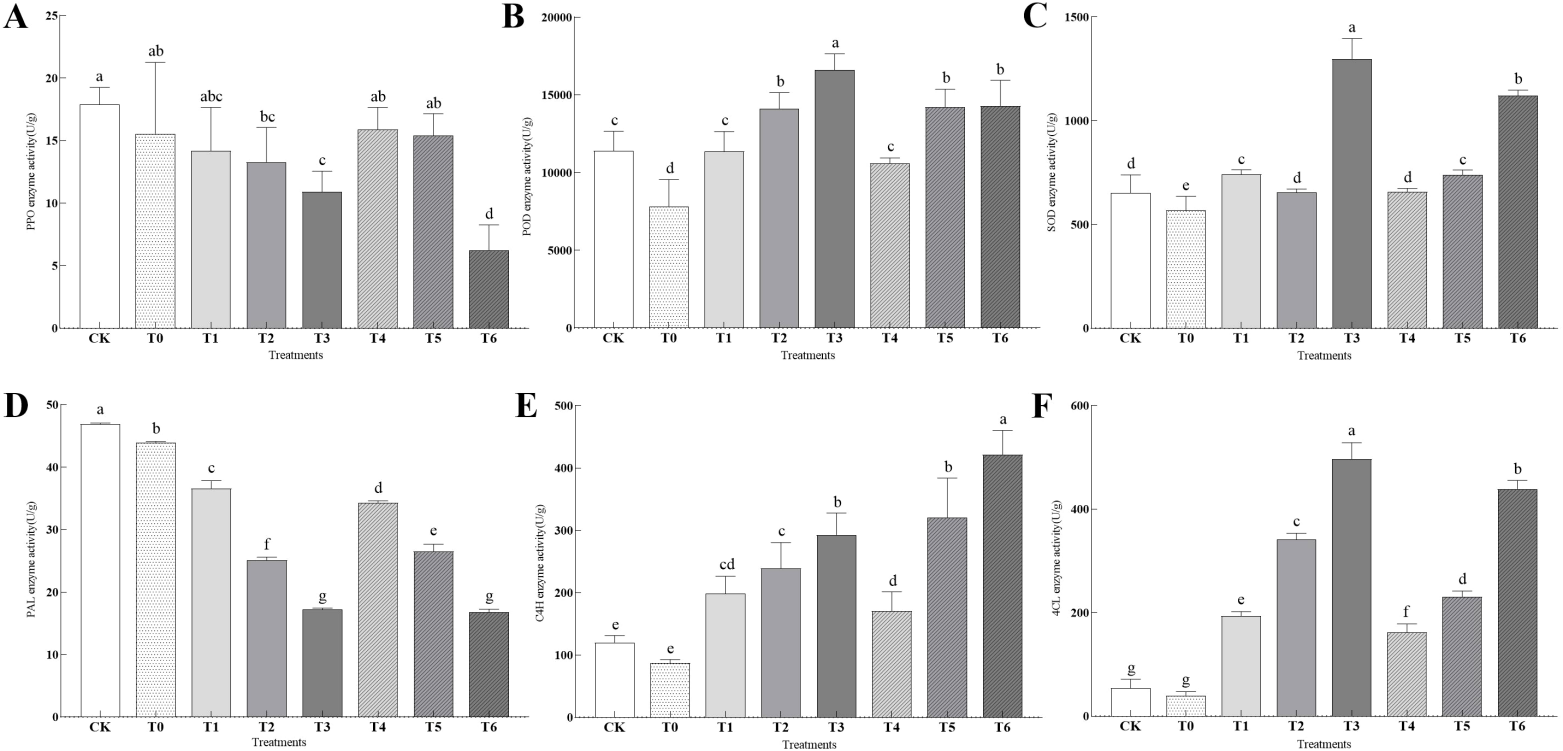
**(A–F)** represent the effects of different treatments on the activities of PPO, POD, SOD, PAL, 4CL, and C4H enzymes, respectively.

As shown in **Figure 9**, all treatments inhibited the activity of PPO, among which T6 treatment had the strongest inhibitory effect, which was 65.10% lower than that of CK; the activities of POD and SOD were significantly promoted, and T3 treatment performed the best, increasing by 45.68% and 99.45% compared to CK, respectively. Regarding enzymes in the phenylpropane pathway: the activity of PAL was most strongly inhibited by T6 treatment, which was 64.25% lower than that of CK; the activities of C4H and 4CL were significantly promoted. T6 treatment had the highest C4H activity, which was 250.89% higher than that of CK, and T3 treatment had the highest 4CL activity, which was 814.03% higher than that of CK.

### 3.8 Genes in the phenylpropane metabolic pathway

**Figure 10:**
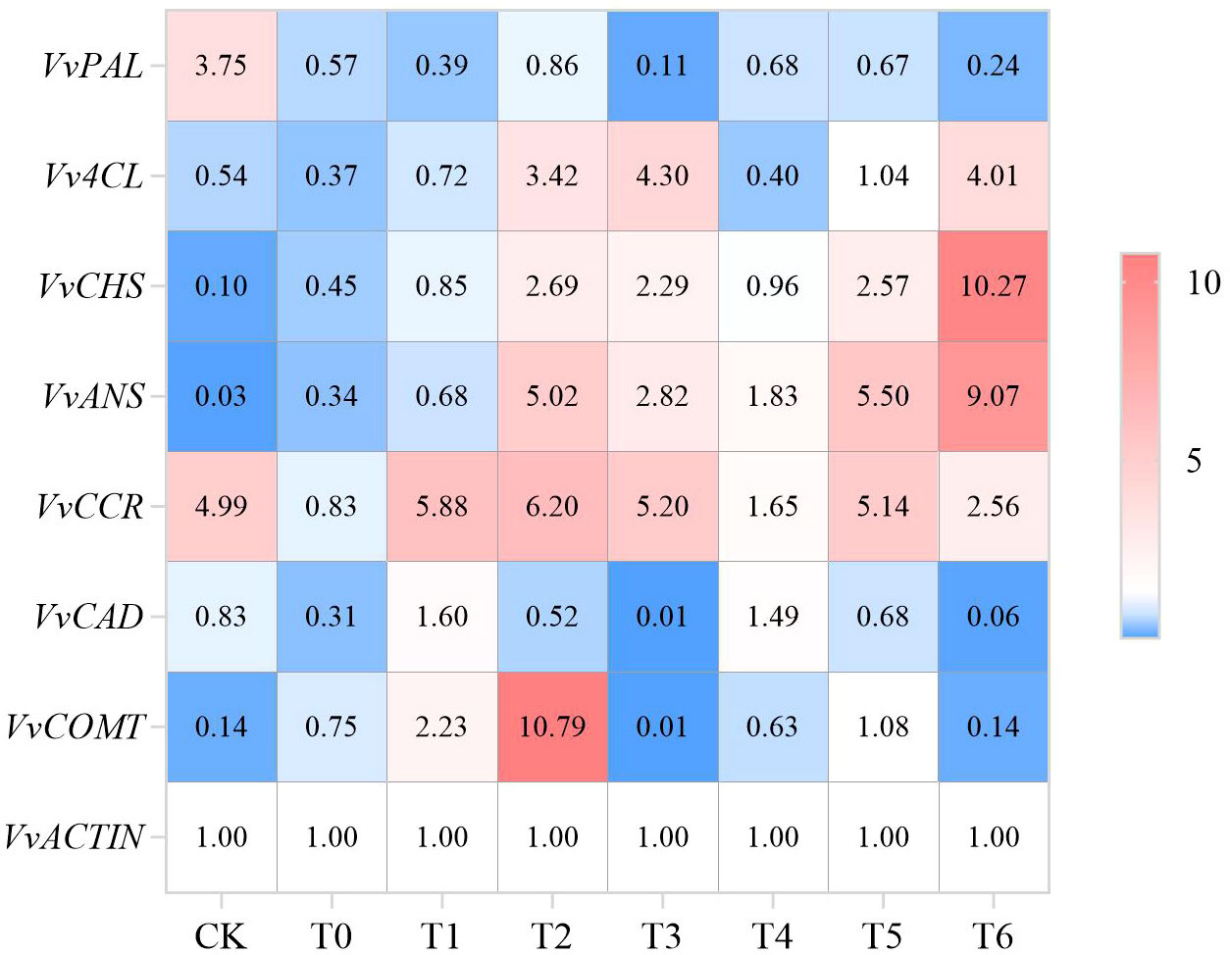
Effects of different treatments on the relative expression of key enzyme genes in the phenylpropanoid metabolic pathway in grape peel.

As shown in **Figure 10**, the reference gene *VvACTIN* was stably expressed in all treatments, providing a reliable standardization basis for quantitative results. Key genes showed differential expression patterns: *VvPAL* (phenylalanine ammonia-lyase gene) was significantly inhibited by CA and PC, and T3 treatment down-regulated it by 97.07% compared to CK; *Vv4CL* (4-coumarate-CoA ligase gene) was strongly induced by CA, and T3 treatment up-regulated it by 6.96-fold compared to CK; *VvCHS* (chalcone synthase gene) and *VvANS* (anthocyanidin synthase gene) were most significantly induced by high-concentration PC, and T6 treatment up-regulated them by 101.70-fold and 301.33-fold compared to CK, respectively, indicating that PC can promote anthocyanin synthesis. The expression of lignin-related genes (*VvCCR*, *VvCAD*, *VvCOMT*) was significantly different, among which *VvCOMT* (caffeic acid-O-methyltransferase gene) was most strongly induced by T2 treatment (75.86-fold up-regulation compared to CK). In summary, CA and PC affect grape quality and storability by regulating different genes in the phenylpropane pathway, and T3 and T6 treatments have the most significant regulatory effects.

### 3.9 Comprehensive evaluation by entropy-weighted TOPSIS

To avoid subjective bias and quantify the treatment effects, this study used the entropy weight TOPSIS method to perform a comprehensive evaluation of multiple indicators (quality and storability), with calculation details and full tables shown in **Supplementary Tables 1 and 2**. As shown in **Table 6**, in terms of fruit quality, treatment T3 had the best overall effect, followed by T6, while T4 had the poorest effect, close to that of T0. As shown in **Table 7**, in terms of storability, treatment T6 performed the best, followed by T3, while T4 and T1 performed poorly. This comprehensive ranking confirms that high-concentration CA (T3) and PC (T6) are the optimal treatment schemes, with T3 being more beneficial for quality improvement and T6 more advantageous for enhancing storability.

**Table 6:** Fruit quality index TOPSIS evaluation and calculation results.

Experimental treatments	The distance of the positive ideal solution	The distance of the negative ideal solution	Relative proximity	Sort the results
CK	0.168	0.063	0.271	6
T0	0.162	0.055	0.254	7
T1	0.121	0.095	0.44	5
T2	0.09	0.118	0.567	3
T3	0.072	0.157	0.686	1
T4	0.157	0.047	0.229	8
T5	0.113	0.1	0.471	4
T6	0.072	0.155	0.684	2

**Table 7:** Fruit storability index TOPSIS evaluation and calculation results.

Experimental treatments	The distance of the positive ideal solution	The distance of the negative ideal solution	Relative proximity	Sort the results
CK	0.104	0.061	0.371	5
T0	0.102	0.059	0.368	6
T1	0.096	0.054	0.359	7
T2	0.072	0.075	0.511	4
T3	0.072	0.097	0.572	2
T4	0.094	0.051	0.352	8
T5	0.07	0.074	0.515	3
T6	0.072	0.098	0.576	1

## 4 Discussion

### 4.1 Effects of different treatments on grape fruit quality

TSS is a core indicator for evaluating fruit sweetness and overall quality (Nie et al., 2014). Sugar accumulation increases intracellular solute concentration and enhances cell turgor, thereby improving the structural compactness of fruits (Li M et al., 2016). Consistent with the results of this study, both CA and PC treatments significantly promoted TSS accumulation, with the high-concentration PC treatment (T6) exhibiting the most prominent effect (6.88% - 14.67% higher than the control group, CK). This observation can be mechanistically linked to the regulation of sugar metabolism: CA and PC may inhibit the activity of sugar-decomposing enzymes to reduce sugar consumption, while simultaneously activating key enzymes involved in sugar synthesis (e.g., sucrose synthase), thereby promoting the production and accumulation of soluble sugars in fruits (Li J et al., 2024). For PC specifically, potassium ions are hypothesized to act as activators of cell membrane sucrose-H^+^ symporters (Wu et al., 2024), which may accelerate the transport of sucrose into cells to enhance fruit sweetness (Kalinin et al., 2001). This synergistic effect of sugar accumulation and improved puncture characteristics collectively contributes to the enhancement of fruit quality, though direct evidence for potassium-mediated sugar transporter activation requires further validation.

TA content is closely correlated with fruit palatability (Zhang et al., 2017). During fruit storage, organic acids are gradually consumed via respiratory metabolism (Li S et al., 2024), and CA/PC treatments may modulate this process by regulating the activity of enzymes involved in organic acid metabolism (e.g., malate dehydrogenase), leading to a reduction in TA content (Zhang et al., 2022). As observed in this study, TA content decreased with prolonged storage across most treatments, with distinct stage-specific differences between CA and PC: high-concentration CA resulted in the lowest TA content during the early storage stage (0–48 h, 25.40% - 36.82% lower than CK), while high-concentration PC dominated in the late storage stage (48–96 h, 13.74% - 31.83% lower than CK). This stage-specific variation may reflect differential impacts of CA and PC on fruit respiratory metabolism and organic acid metabolic enzyme activities. When combined with the accumulation of TSS, the reduction in TA optimizes the solid-acid ratio of fruits, further improving their taste quality.

Antioxidant substances, including total phenols, flavonoids, anthocyanins, and ascorbic acid, play crucial roles in regulating fruit quality and stress resistance, with high-concentration CA and PC treatments driving significant increases in their contents. Total phenol content was positively correlated with fruit puncture strength, which may be attributed to the involvement of phenolic substances in cell wall reinforcement (Martim et al., 2024). Additionally, the synthesis of phenolic compounds relies on sugar-derived precursors (Guan et al., 2024), forming a synergistic regulatory loop between sugar metabolism and phenolic accumulation. Flavonoids and anthocyanins, key determinants of fruit color, reached peak levels in the high-concentration PC treatment at 96 h (145.89% and 723.27% higher than CK, respectively), a trend that can be directly linked to the significant up-regulation of *VvCHS* (101.70-fold higher than CK) and *VvANS* (301.33-fold higher than CK) genes in the phenylpropanoid metabolic pathway. Ascorbic acid content increased with treatment concentration and may indirectly maintain cell turgor by regulating fruit osmotic potential (Ajila et al., 2023), establishing a functional connection between antioxidant metabolism and fruit physical quality.

The activity of PAL and the expression trend of its homologous gene *VvPAL* showed an opposite change to those of C4H and 4CL. Specifically, the high-concentration PC treatment reduced PAL activity by 64.25% compared with the control (CK), but significantly enhanced C4H and 4CL activities, increasing them by 250.89% and 814.03% respectively relative to CK. This difference may reflect the redistribution of the phenylpropanoid metabolic flux—metabolic resources are shifted from lignin synthesis to the synthesis of antioxidant substances such as flavonoids and anthocyanins. Consequently, fruit coloring and antioxidant capacity are improved while excessive lignification is avoided.

### 4.2 Effects of different treatments on the storability of grape berries

#### 4.2.1 Regulatory mechanism of fruit softening

Fruit puncture characteristics (including peel puncture strength and flesh firmness) are direct indicators of cell wall integrity (Xie et al., 2024)), and their maintenance by high-concentration CA and PC treatments can be mechanistically attributed to the suppression of cellulase and pectinase activities. These two hydrolases are known to decompose cellulose and pectin components in the cell wall, leading to fruit softening (Lu et al., 2022). In the present study, high-concentration CA reduced pectinase activity by 57.83% - 64.39% compared to CK, while medium-concentration PC exhibited the strongest inhibitory effect on cellulase (82.62% - 92.28% lower than CK). This enzyme-level regulation effectively slowed the rate of cell wall degradation, enhancing the pressure-bearing capacity of fruits during puncture and maintaining their physical structural stability.

A key “lignin paradox”—a novel and intriguing finding of this study—was observed: high-concentration PC significantly improved flesh firmness (156.28% higher than CK at 96 h) while reducing lignin content by 37.36% compared to CK at the same time point. This result contradicts the conventional view that lignin accumulation is positively correlated with fruit firmness. The underlying mechanism may involve the redirection of phenylpropanoid metabolic flux: the inhibition of PAL activity (a rate-limiting enzyme in lignin synthesis) by high-concentration PC shifts metabolic resources from lignin deposition to the synthesis of antioxidant compounds (e.g., anthocyanins, flavonoids). These antioxidants protect cell membrane integrity and reduce oxidative damage to cell wall components, thereby maintaining fruit firmness via a non-lignin-dependent pathway.

In this study, HP-β-CD was used as a solubilizer for the CA treatment, whereas no solubilizer was employed in the PC treatment. This discrepancy may introduce biases into the direct comparison of the effects between CA and PC treatments. Therefore, a control group treated with HP-β-CD alone (T0) was established in this study to isolate its independent effect. Nevertheless, the available data consistently indicate that PC treatment exhibits superior performance in enhancing fruit storability. For example, at the same concentration, PC treatment outperforms CA in maintaining the ratio of peel elasticity to crispness, which may be associated with potassium ions improving cell wall elasticity through osmotic regulation (Kalinin et al., 2001). This might be partially attributed to potassium ion-mediated osmotic adjustment or transporter activation, which awaits further experimental verification.

#### 4.2.2 Mechanism for the prevention and control of falling grains

Berry drop rate is closely associated with the mechanical stability of the grape stems–pedicel system, and high-concentration CA and PC treatments significantly reduced fruit drop by maintaining the structural integrity and mechanical properties of this system. At 96 h the terminal storage stage with practical application significance, high-concentration CA achieved the lowest drop rate (77.13% lower than CK), which was attributed to its superior ability to inhibit grape stems browning (77.75% lower than CK at 96 h) and preserve the structural integrity of pedicels. In contrast, high-concentration PC enhanced the tensile properties of the grape stems–pedicel system: the maximum tensile strength was 173.24% higher than CK at 96 h, and tensile energy was significantly increased. These mechanical improvements are mechanistically linked to the inhibition of cellulase and pectinase activities, slowing the degradation of cellulose and hemicellulose (An et al., 2024); and potassium-mediated regulation of cell osmotic pressure, improving cell wall elasticity (Shane and Karin, 2021).

Fruit brush traits (length and thickness) further contributed to the reduction in berry drop. Longer fruit brush (79.37% longer than CK in high-concentration PC at 96 h) dispersed the gravitational force of berries to the grape stems via the lever principle, reducing pressure at the grape stems connection site (Yan et al., 2021). Meanwhile, thicker fruit brush (69.03% thicker than CK in high-concentration CA at 96 h) strengthened mechanical support through their well-developed vascular bundle system. These traits collectively maintained the stability of the grape stems–pedicel system: thicker fruit brush not only formed high-efficiency channels for nutrient transport but also reinforced the connection between berries and the grape stems, while higher tensile deformation (123.31% higher than CK in high-concentration CA at 96 h) avoided structural fracture via uniform stress dispersion (Zhu et al., 2022), enabling adaptation to fluctuations in the storage environment and reducing mechanical damage.

#### 4.2.3 Berry rot inhibition mechanism

High-concentration CA and PC treatments effectively reduced fruit decay rate by regulating microbial community structure and the oxidative defense system. Their effects on microorganisms manifest as complex niche dynamics rather than simple inhibition: high-concentration CA treatment could inhibit *Alternaria* in the short term, but the abundance of *Penicillium* rebounded in the late storage period; in contrast, high-concentration PC treatment achieved sustained inhibition of *Fusarium* (abundance range: 0.38%~1.08%) and induced niche replacement of *Penicillium* by *Aspergillus* (the abundance of *Aspergillus* reached 22.69% at 96 h). Potassium ions may increase the permeability of fungal cell membranes, thereby enhancing the inhibitory effect of cinnamic acid. This is consistent with the conclusion by Liao that “CA exerts antibacterial effects by damaging fungal cell membranes” (Liao et al., 2021), but direct evidence for this synergistic effect is lacking and requires further verification.

### 4.3 Integrated mechanistic model

**Figure 11:**
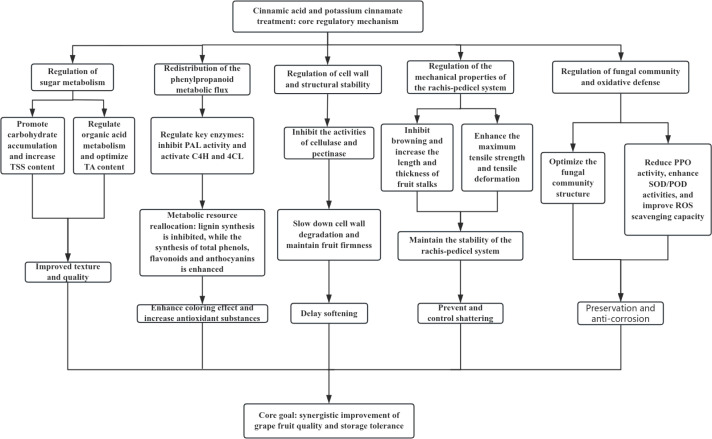
Conceptual framework diagram of CA and PC processing regulation mechanism.

To integrate the multifaceted effects of CA and PC treatments, a conceptual framework for their regulatory mechanisms is proposed (**Figure 11**):

High-concentration CA: Primarily optimizes fruit quality by reducing TA content during the early storage stage, promoting anthocyanin synthesis via *VvCHS*/*VvANS* up-regulation, and maintaining grape stems browning resistance.

High-concentration PC: Excels in enhancing fruit storability by inhibiting cellulase and pectinase activities to maintain firmness, redirecting phenylpropanoid metabolic flux to resolve the “lignin paradox,” and stabilizing microbial community niches. The potassium ion in PC is hypothesized to contribute to these effects via osmotic regulation or transporter activation, though this requires experimental validation.

The storage duration of this study was relatively short (only 0–96 h), which fails to reflect the effects of CA/PC treatments on long-term storage (e.g., 7 days and longer). This limitation restricts the generalizability of the research conclusions in scenarios involving extended supply chains. Additionally, the research results are only applicable to ‘Kyoho’ grapes, and their applicability to other grape cultivars (such as ‘Red Globe’ and ‘Thompson Seedless’) remains unclear.

In future studies, the following aspects should be addressed: first, extend the storage duration to verify the long-term stability of the effects of CA/PC treatments, with specific focus on their impacts on fruit rot and sensory quality. Second, validate the effects of 10 mmol·L^-1^ CA/PC treatments in other grape cultivars to evaluate cultivar adaptability. Additionally, molecular experiments (e.g., sugar transporter gene knockout, potassium ion flux detection) should be conducted to verify hypothetical mechanisms, such as potassium-mediated sugar transport and ion-phenolic synergistic antibacterial effects.

## 5 Conclusion

This study aimed to address the postharvest issues of softening, berry drop, and decay in ‘Kyoho’ grapes by investigating the regulatory effects of CA and PC at different concentrations. During the 0–96 h storage period, the 10 mmol·L^-1^ CA treatment exhibited stage-specific quality improvement effects: it significantly increased TSS content, optimized the solid-acid ratio, and enhanced antioxidant levels, with a more pronounced effect on reducing TA content in the early storage stage (0–48 h). The 10 mmol·L^-1^ PC treatment effectively delayed fruit softening by inhibiting cellulase and pectinase activities to slow cell wall degradation, thereby maintaining peel puncture strength, flesh firmness, and brittleness ratio. Meanwhile, high-concentration CA/PC treatments both reduced the berry drop rate in a concentration-dependent manner: CA alleviated grape stems browning and enhanced pedicel tensile resistance, while PC increased the length and thickness of the fruit brush. Additionally, these high-concentration treatments reduced decay by inhibiting the abundance of pathogenic bacteria, with PC showing a more durable antibacterial effect; PC also regulated antioxidant enzyme activities to delay senescence. In practical applications, foliar spraying of 10 mmol·L^-1^ 7 days before harvest is recommended for prioritizing fruit quality improvement, while 10 mmol·L^-1^ PC is more favorable for enhancing storability.

In conclusion, pre-harvest application of 10 mmol·L^-1^ CA and 10 mmol·L^-1^ PC provides effective solutions for improving the quality and storability of ‘Kyoho’ grapes, respectively, with concentration- and time-dependent effects. These treatments are expected to reduce postharvest losses and enhance the economic benefits of the industry, but further verification of their long-term effects and cultivar adaptability is required before large-scale application.

## Data Availability

The original contributions presented in the study are included in the article/**Supplementary Material**. Further inquiries can be directed to the corresponding authors.

## References

[B1] Ajila C. G. E. Lupino G. P. Bereta L. M. G. D. Dos R. A. R. (2023). Physiological and biochemical roles of ascorbic acid on mitigation of abiotic stresses in plants. Plant Physiol. Biochem. 202, 107970. doi: 10.1016/J.PLAPHY.2023.107970, PMID: 37625254

[B2] An Y. Liu X. Zhan C. Zhao Y. Zhang J. Li M . (2024). Investigations of the inhibition of lignin on enzymatic hydrolysis of cellulase. J. Henan Agric. Univ. 58, 23–34. doi: 10.16445/j.cnki.1000-2340.20231101.001

[B3] Cao J. Jiang W. Zhao Y. (2019). Guidance for postharvest physiological and biochemical experiments of fruits and vegetables (Beijing, China: China Light Industry Press).

[B4] Chen Z. (2019). Problems and solutions of grape production in fu’an city. Horticulture Seed 39, 41–43. doi: 10.16530/j.cnki.cn21-1574/s.2019.06.013

[B5] Guan Y. Wang Y. Yuan N. Saren G. Ke F. Hu W . (2024). Biosynthetic mechanism of phenolic substances and theirantioxidant activity in fresh-cut fruits and vegetables: a review. J. Chin. Institute Food Sci. Technol. 24, 397–406. doi: 10.16429/j.1009-7848.2024.02.036

[B6] Hu Y. Tian S. Wang C. Wang R. Ma C. (2024). Effect of preharvest cinnamic acid treatment on storability of ‘kyoho’ grapes. Sino-Overseas Grapevine Wine, 26–31. doi: 10.13414/j.cnki.zwpp.2024.04.004

[B7] Ji X. Wang B. Wang X. Shi X. Liu P. Liu F . (2018). Effects of different color paper bags on aroma development of kyoho grape berries. J. Integr. Agric. 18, 70–82. doi: 10.1016/S2095-3119(18)62008-8

[B8] Kalinin V. A. Orlova O. V. Opritov V. A. (2001). Kinetics of δψ-dependent sucrose transport in plasma membrane vesicles from higher plant cells. Russian J. Plant Physiol. 48, 300–307. doi: 10.1023/A:1016602113980

[B9] Koki Y. Takashi T. Junki T. Yoshiyuki I. Daisuke N. Keiji T. (2023). Characterization, preparation, and promotion of plant growth of 1,3-diphenylurea/β-cyclodextrin derivatives inclusion complexes. Acs Omega. 8, 34972–34981. doi: 10.1021/ACSOMEGA.3C04428, PMID: 37779935 PMC10536069

[B10] Lai C. Pan H. Zhang J. Wang Q. Gao H. Chen Y . (2019). Selection and validation of reference genes forquantitative real-time polymerase chain reaction(qrt.pcr)after different shoot pinching treatments on grape. Acta Agriculturae Universitatis Jiangxiensis 41, 890–900. doi: 10.13836/j.jjau.2019102

[B11] Li M. Deng Q. Lu X. Liu J. Cheng X. (2016). Sugar accumulation and sucrose-metabolizing enzymesactivities of ‘manicure finger grape. J. Northwest Agric. Forestry Univ. (Natural Sci. Edition) 44, 185–190. doi: 10.13207/j.cnki.jnwafu.2016.08.027

[B12] Li J. Hu Y. Hu J. Xie Q. Chen X. Qi X . (2024). Sucrose synthase: an enzyme with multiple roles in plant physiology. J. Plant Physiol. 303, 154352. doi: 10.1016/J.JPLPH.2024.154352, PMID: 39332324

[B13] Li X. Jin J. Li B. Wang C. Dong X. . (2016). Community structure and temporal dynamics of fungi incuticle and core of bagging and un-bagging apple fruits. Mycosystema 35, 927–938. doi: 10.13346/j.mycosystema.150091

[B14] Li S. Liu G. Ren H. Zhou S. Li J. Feng M . (2024). Response of organic acid metabolism in the young and ripening grape berries *in vitro* to ambient temperature. Plant Cell Tissue Organ Culture (Pctoc) 157, 1–12. doi: 10.1007/S11240-024-02729-1

[B15] Li F. Wang S. Gu S. Cheng D. Gu H. Li M . (2020). Effects of foliar application of aba and pdj on the coloration and quality of ‘kyoho’ grape berry. J. Fruit Sci. 37, 362–370. doi: 10.13925/j.cnki.gsxb.20190551

[B16] Liao J. Hu W. Long Y. Zhao M. Yang X. Li Y . (2021). Application of electron microscopy in the study of postharvestpreservation of berries. Sci. Technol. Food Industry 42, 356–361. doi: 10.13386/j.issn1002-0306.2020060062

[B17] Liu J. (2022). Effects of different treatments on surface fungus and storage quality of ‘jimei’ cherry. Xianyang, Shaanxi, China: Northwest A&F University.

[B18] Lu L. Ma Y. Song G. Zhang H. Gu X. Xiao J . (2022). Changes of polysaccharide content and pectindegradation related enzyme activities in cell wall duringsoftening of kiwifruit. Acta Agriculturae Zhejiangensis 34, 2648–2658. doi: 10.3969/j.issn.1004-1524

[B19] Martim D. B. Brilhante A. J. V. C. Lima A. R. Paixão D. A. A. Junior J. M. Kashiwagi F. M . (2024). Resolving the metabolism of monolignols and other lignin-related aromatic compounds in xanthomonas citri. Nat. Commun. 15, 7994. doi: 10.1038/S41467-024-52367-6, PMID: 39266555 PMC11393088

[B20] Nie J. Li J. Xu G. Li H. Yan Z. Kuang L . (2014). Screening suitable juicing methods for determination of total soluble solids content in fruits. Storage Process 14, 62–64. doi: 10.3969/i.issn.1009-6221.2014.05.012

[B21] Pablo C. Eva S. M. Rafael T. Carolina R. Diego L. Gema B . (2013). Thermotolerance responses in ripening berries of vitis vinifera l. Cv muscat hamburg. Plant Cell Physiol. 54, 1200–1216. doi: 10.1093/pcp/pct071, PMID: 23659918

[B22] Ruwizhi N. Aderibigbe B. A. (2020). Cinnamic acid derivatives and their biological efficacy. Int. J. Mol. Sci. 21, 5712. doi: 10.3390/ijms21165712, PMID: 32784935 PMC7460980

[B23] Shane A. Karin N. (2021). Mitochondrial osmoregulation in evolution, cation transport and metabolism. Biochim. Et Biophys. Acta (Bba) - Bioenergetics 1862, 148368. doi: 10.1016/J.BBABIO.2021.148368, PMID: 33422486

[B24] Shang J. Wu W. Ma Y. (2022). Phenylpropanoid metabolism pathway in plants. Chin. J. Biochem. Mol. Biol. 38, 1467–1476. doi: 10.13865/j.cnki.cjbmb.2022.03.1604

[B25] Su Z. Wang X. Xuan X. Sheng Z. Jia H. Emal N . (2021). Characterization and action mechanism analysis of vvmir156b/c/d-vvspl9 module responding to multiple-hormone signals in the modulation of grape berry color formation. Foods 10, 896. doi: 10.3390/FOODS10040896, PMID: 33921800 PMC8073990

[B26] Tyagi K. Maoz I. Vinokur Y. Rodov V. Lewinsohn E. Lichter A . (2020). Enhancement of table grape flavor by postharvest application of monoterpenes in modified atmosphere. Postharvest Biol. Technol. 159, 111018. doi: 10.1016/j.postharvbio.2019.111018

[B27] Wang D. Zhang M. Yue Z. Zhou H. (2021). Chlorogenic acid treatment induced resistance topostharvest gray mold on apples. Food Sci. 42, 177–183. doi: 10.7506/spkx1002-6630-20200514-156

[B28] Wang Y. Pang K. Zhu B. Fan S. Hu W. (2007). Effect of mechanical damage on the physiology and biochemistry in fuji apple. Food and Fermentation Industries, 58–62. doi: 10.13995/j.cnki.11-1802/ts.2007.07.018

[B29] Wu K. Hu C. Liao P. Hu Y. Sun X. Tan Q . (2024). Potassium stimulates fruit sugar accumulation by increasing carbon flow in citrus sinensis. Horticulture Res. 11, e240. doi: 10.1093/HR/UHAE240, PMID: 39512779 PMC11540757

[B30] Xie Y. Chen L. Zhang M. Hu Z. (2024). Research progress on fruit firmness and crack resistance mechanism in fruits and vegetables. Plant Physiol. J. 60, 1514–1523. doi: 10.13592/j.cnki.ppj.300273

[B31] Yan D. Wang J. Luo L. Liu W. Wei H. Wang J . (2021). Vibration shedding characteristics of the grapes under the excitation of broken stems and experimental research. Trans. Chin. Soc. Agric. Eng. 37, 31–40. doi: 10.11975/i.issn.1002-6819.2021.22.004

[B32] Yu P. Meng X. Yu Y. Yang Y. (2023). Effect of preharvest spray of diethyl aminoethyl hexanoate on postharvest fruit quality and reactiveoxygen species metabolismin ‘kyoho’ grape. Food Sci. 44, 182–188. doi: 10.7506/spkx1002-6630-20220124-241

[B33] Zhang L. (2020). Changes of polyphenols in the quality factors of different grape varieties under high temperature stress and their antioxidant resistance were studied. Xi'an, Shaanxi, China: Shaanxi Normal University.

[B34] Zhang S. Li G. Guo W. Lian Y. Geng X. Ju Y . (2017). Effect of 1-mcp on fruit hardness, titratable acid and sod activity of ‘xizhoumi no.25’ during storage. China Cucurbits Vegetables 30, 17–20. doi: 10.16861/j.cnki.zggc.2017.0166

[B35] Zhang L. Ma B. Wang C. Chen X. Ruan Y. Yuan Y . (2022). Mdwrky126 modulates malate accumulation in apple fruit by regulating cytosolic malate dehydrogenase (mdmdh5). Plant Physiol. 188, 2059–2072. doi: 10.1093/PLPHYS/KIAC023, PMID: 35078249 PMC8968328

[B36] Zhu J. Zhu D. Wang L. Xue K. Liao J. Zhang S . (2022). Effects of compression damage on mechanical behavior and quality attributes of apple fruit:technical papers. Food Sci. Technol. Res. 28, 53–65. doi: 10.3136/FSTR.FSTR-D-21-00178

