# Supplementary material for: Pre-harvest cinnamic acid and potassium cinnamate improve postharvest quality and storability of ‘Kyoho’ grapes
Source: Front Plant Sci. 2026 Jan 12;16:1713059. doi: 10.3389/fpls.2025.1713059 (PMC12833327; doi:10.3389/fpls.2025.1713059)
Supplement: Supplementary file 1 [file Table1.docx]

Supplementary Material

# Supplementary Tables

For more information on Supplementary Material and for details on the different file types accepted, please see [here](https://www.frontiersin.org/guidelines/author-guidelines#supplementary-material).

Table 1 Summary of the weight calculation results of fruit quality entropy method

| Storage time（d） | Experimental metrics | Information entropy | Information utility value d | Weight coefficient w |
| --- | --- | --- | --- | --- |
| 0 h | Soluble solids | 0.9877 | 0.0123 | 3.04% |
|  | Titratable acids | 0.985 | 0.015 | 3.68% |
|  | Total phenols | 0.9867 | 0.0133 | 3.27% |
|  | Flavonoids | 0.984 | 0.016 | 3.94% |
|  | Anthocyanidin | 0.9853 | 0.0147 | 3.61% |
|  | Ascorbic acid | 0.9848 | 0.0152 | 3.74% |
| 24 h | Soluble solids | 0.9829 | 0.0171 | 4.20% |
|  | Titratable acids | 0.9916 | 0.0084 | 2.06% |
|  | Total phenols | 0.9894 | 0.0106 | 2.60% |
|  | Flavonoids | 0.988 | 0.012 | 2.95% |
|  | Anthocyanidin | 0.9897 | 0.0103 | 2.53% |
|  | Ascorbic acid | 0.9882 | 0.0118 | 2.91% |
| 48 h | Soluble solids | 0.9906 | 0.0094 | 2.30% |
|  | Titratable acids | 0.9867 | 0.0133 | 3.27% |
|  | Total phenols | 0.9795 | 0.0205 | 5.04% |
|  | Flavonoids | 0.9876 | 0.0124 | 3.06% |
|  | Anthocyanidin | 0.9857 | 0.0143 | 3.52% |
|  | Ascorbic acid | 0.9832 | 0.0168 | 4.12% |
| 72 h | Soluble solids | 0.9873 | 0.0127 | 3.12% |
|  | Titratable acids | 0.9905 | 0.0095 | 2.34% |
|  | Total phenols | 0.9805 | 0.0195 | 4.80% |
|  | Flavonoids | 0.9868 | 0.0132 | 3.24% |
|  | Anthocyanidin | 0.9862 | 0.0138 | 3.39% |
|  | Ascorbic acid | 0.9829 | 0.0171 | 4.22% |
| 96 h | Soluble solids | 0.99 | 0.01 | 2.47% |
|  | Titratable acids | 0.988 | 0.012 | 2.96% |
|  | Total phenols | 0.989 | 0.011 | 2.71% |
|  | Flavonoids | 0.9851 | 0.0149 | 3.66% |
|  | Anthocyanidin | 0.987 | 0.013 | 3.19% |
|  | Ascorbic acid | 0.9835 | 0.0165 | 4.07% |

Table 2 Summary of the weight results of fruit storability entropy method

| Storage time（d） | Experimental metrics | Information entropy | Information utility value | | Weight coefficient w | | |
| --- | --- | --- | --- | --- | --- | --- | --- |
| 0 h | Peel puncture strength | 0.9902 | 0.0098 | | 1.25% | | |
|  | The elasticity of the peel | 0.988 | 0.012 | | 1.52% | | |
|  | Average flesh hardness | 0.9827 | 0.0173 | | 2.20% | | |
|  | Brittleness ratio | 0.9909 | 0.0091 | | 1.15% | | |
|  | Grain drop rate | 0.9866 | 0.0134 | | 1.71% | | |
|  | Degree of browning of fruit peduncles | 0.9899 | 0.0101 | | 1.28% | | |
|  | Maximum tensile strength | 0.9906 | 0.0094 | | 1.20% | | |
|  | Tensile deformation | 0.9877 | | 0.0123 | | 1.56% | |
|  | Stretch energy | 0.984 | | 0.016 | | 2.04% | |
|  | Fruit brush length | 0.9903 | | 0.0097 | | 1.23% | |
|  | Fruit brush thickness | 0.9905 | | 0.0095 | | 1.21% | |
|  | Lignin content of fruits | 0.9891 | | 0.0109 | | 1.39% | |
| 24 h | Peel puncture strength | 0.9884 | | 0.0116 | | 1.48% | |
|  | The elasticity of the peel | 0.988 | | 0.012 | | 1.53% | |
|  | Average flesh hardness | 0.9869 | | 0.0131 | | 1.67% | |
|  | Brittleness ratio | 0.9854 | | 0.0146 | | 1.86% | |
|  | Grain drop rate | 0.9887 | | 0.0113 | | 1.43% | |
|  | Degree of browning of fruit peduncles | 0.9883 | | 0.0117 | | 1.49% | |
|  | Maximum tensile strength | 0.9895 | | 0.0105 | | 1.34% | |
|  | Tensile deformation | 0.99 | | 0.01 | | 1.27% | |
|  | Stretch energy | 0.9879 | | 0.0121 | | 1.53% | |
|  | Fruit brush length | 0.9921 | | 0.0079 | | 1.01% | |
|  | Fruit brush thickness | 0.9904 | | 0.0096 | | 1.22% | |
|  | Lignin content of fruits | 0.9886 | | 0.0114 | | 1.45% | |
| 48 h | Peel puncture strength | 0.9862 | | 0.0138 | | 1.76% | |
|  | The elasticity of the peel | 0.9867 | | 0.0133 | | 1.69% | |
|  | Average flesh hardness | 0.9864 | | 0.0136 | | 1.73% | |
|  | Brittleness ratio | 0.9915 | | 0.0085 | | 1.08% | |
|  | Grain drop rate | 0.9892 | | 0.0108 | | 1.38% | |
|  | Rotten fruit rate | 0.9751 | | 0.0249 | | 3.17% | |
|  | Degree of browning of fruit peduncles | 0.9827 | | 0.0173 | | 2.20% | |
|  | Maximum tensile strength | 0.9867 | | 0.0133 | | 1.69% | |
|  | Tensile deformation | 0.9839 | | 0.0161 | | 2.05% | |
|  | Stretch energy | 0.9899 | | 0.0101 | | 1.28% | |
|  | Fruit brush length | 0.984 | | 0.016 | | 2.03% | |
|  | Fruit brush thickness | 0.9906 | | 0.0094 | | 1.20% | |
|  | Lignin content of fruits | 0.989 | | 0.011 | | 1.40% | |
| 72 h | Peel puncture strength | 0.9883 | | 0.0117 | | 1.49% | |
|  | The elasticity of the peel | 0.9908 | | 0.0092 | | 1.17% | |
|  | Average flesh hardness | 0.9858 | | 0.0142 | | 1.81% | |
|  | Brittleness ratio | 0.9899 | | 0.0101 | | 1.28% | |
|  | Grain drop rate | 0.9854 | | 0.0146 | | 1.86% | |
|  | Rotten fruit rate | 0.9861 | | 0.0139 | | 1.77% | |
|  | Degree of browning of fruit peduncles | 0.9834 | | 0.0166 | | 2.11% | |
|  | Maximum tensile strength | 0.9886 | | 0.0114 | | 1.45% | |
|  | Tensile deformation | 0.987 | | 0.013 | | 1.66% | |
|  | Stretch energy | 0.9876 | | 0.0124 | | 1.58% | |
|  | Fruit brush length | 0.9835 | | 0.0165 | | 2.10% | |
|  | Fruit brush thickness | 0.99 | | 0.01 | | 1.27% | |
|  | Lignin content of fruits | 0.9887 | | 0.0113 | | 1.44% | |
| 96 h | Peel puncture strength | 0.9878 | | 0.0122 | | 1.55% | |
|  | The elasticity of the peel | 0.9918 | | 0.0082 | | 1.04% | |
|  | Average flesh hardness | 0.9885 | | 0.0115 | | 1.46% | |
|  | Brittleness ratio | 0.9857 | | 0.0143 | | 1.82% |  |
|  | Grain drop rate | 0.9851 | | 0.0149 | | 1.90% |  |
|  | Rotten fruit rate | 0.985 | | 0.015 | | 1.91% |  |
|  | Degree of browning of fruit peduncles | 0.986 | | 0.014 | | 1.78% |  |
|  | Maximum tensile strength | 0.9869 | | 0.0131 | | 1.67% |  |
|  | Tensile deformation | 0.9882 | | 0.0118 | | 1.50% |  |
|  | Stretch energy | 0.987 | | 0.013 | | 1.65% |  |
|  | Fruit brush length | 0.9834 | | 0.0166 | | 2.12% |  |
|  | Fruit brush thickness | 0.9886 | | 0.0114 | | 1.45% |  |
|  | Lignin content of fruits | 0.9885 | | 0.0115 | | 1.47% |  |

**
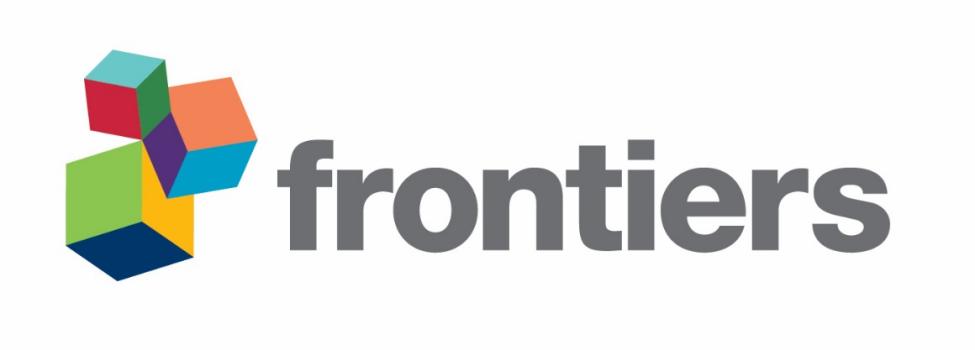
**
